# Evaluation of Darifenacin for T-Cell Acute Lymphoblastic Leukemia: Selective Targeting of the Non-Neuronal Cholinergic System and Lysosomal Cathepsins

**DOI:** 10.3390/ijms27146417

**Published:** 2026-07-19

**Authors:** Luis A. Flores-López, Yoalli Martínez-Pérez, Ignacio De la Mora-De la Mora, Gabriela López-Herrera, Saúl Gómez-Manzo, Itzhel García-Torres, Beatriz Hernández-Ochoa, Sergio Enríquez-Flores

**Affiliations:** 1 SECIHTI-Laboratorio de Biomoléculas y Salud Infantil, Instituto Nacional de Pediatría, Mexico City 04530, Mexico; 2 Escuela de Medicina y Ciencias de la Salud, Tecnológico de Monterrey, Mexico City 14380, Mexico; yoalli.martinez@tec.mx; 3 Laboratorio de Biomoléculas y Salud Infantil, Instituto Nacional de Pediatría, Mexico City 04530, Mexico; ignaciodelamora@ciencias.unam.mx (I.D.l.M.-D.l.M.); garcia.itzhel@gmail.com (I.G.-T.); 4 Laboratorio de Inmunodeficiencias, Instituto Nacional de Pediatría, Mexico City 04530, Mexico; lohegabyqbp@gmail.com; 5 Laboratorio de Bioquímica Genética, Instituto Nacional de Pediatría, Mexico City 04530, Mexico; saulmanzo@ciencias.unam.mx; 6 Laboratorio de Investigación en Ciencias Ómicas y Epidemiología Microbiana, Hospital Infantil de México Federico Gómez, Secretaría de Salud, Mexico City 06720, Mexico; beatrizhb_16@comunidad.unam.mx

**Keywords:** Darifenacin, M3 muscarinic receptor, non-neuronal cholinergic system, T-cell acute lymphoblastic leukemia, selective cytotoxicity, apoptosis, drug repurposing, cathepsin B, cathepsin C, oxidative stress

## Abstract

T-cell acute lymphoblastic leukemia (T-ALL) is an aggressive hematological malignancy that requires novel therapeutic targets and selective repurposed drugs with low systemic toxicity. Here, we evaluated the clinically approved M3 muscarinic receptor antagonist, Darifenacin (DF), against Jurkat and MOLT-4 T-ALL cells, as well as healthy human T lymphocytes, under identical conditions. DF induced selective, concentration-dependent cytotoxicity with IC_50_ values of 26.7 ± 1.07 µM (Jurkat) and 30.5 ± 1.54 µM (MOLT-4), whereas healthy T lymphocytes exhibited an apparent IC_50_ of 197.9 ± 1.29 µM, corresponding to a 6.5–7.4-fold therapeutic window. Mechanistically, DF decreased M3 receptor and choline acetyltransferase expression and increased acetylcholinesterase activity. Convergent multi-assay validation confirmed that this cytotoxicity was driven by activation of the intrinsic mitochondrial apoptotic pathway, as evidenced by increased Bax, decreased Bcl-2, and cleavage of the executioner caspase-3. Furthermore, DF induced glycative stress through the accumulation of methylglyoxal (MG) and advanced glycation end products (AGEs), and selectively inhibited lysosomal Cathepsins B and C. Molecular docking predicted highly favorable molecular binding within the catalytic cavities of these proteases. These findings suggest that DF exhibits selective antileukemic activity by simultaneously disrupting cholinergic signaling, inducing glycative stress, inhibiting cathepsins, and triggering apoptosis. Thus, DF emerges as a promising candidate for multi-target drug repurposing in T-ALL therapy.

## 1. Introduction

### 1.1. Global Burden of Cancer and Childhood Leukemia: Clinical Challenges

Cancer remains one of the leading causes of death worldwide, with global registries reporting approximately 20 million new cases and 9.7 million cancer-related deaths in 2022 [1]. This burden is particularly severe in pediatric populations within low- and middle-income regions such as Latin America and the Caribbean, where nearly 29,000 children are diagnosed annually and approximately 10,000 die from the disease [2]. Consequently, pediatric malignancies constitute a major public health challenge and a leading cause of disease-related mortality, imposing profound physical, psychological, and economic burdens on families [3,4]. Despite advances in pediatric oncology, massive survival disparities persist; while survival rates in high-income countries exceed 80%, they remain close to 30% in resource-limited settings due to delayed diagnoses, treatment abandonment, treatment-related toxicity, and high rates of disease progression [2,5,6,7].

Among these malignancies, leukemia is a dominant global health concern, ranking as the thirteenth most common cancer with 474,519 new cases and over 305,000 deaths annually [1]. Acute lymphoblastic leukemia (ALL) is classified into B-cell ALL (75% of cases) and T-ALL (25% of cases) [8]. Although less frequent, T-ALL presents an aggressive clinical course, frequent chemoresistance, a higher incidence of central nervous system involvement, and a poorer prognosis [8,9]. Patients often present with elevated leukocyte counts, mediastinal masses, and extramedullary disease [10]. Current protocols rely on intensive multi-agent chemotherapy regimens associated with severe acute and long-term toxicities, including cardiotoxicity and neurotoxicity [11,12]. Due to its molecular heterogeneity and genetic alterations affecting *NOTCH1* signaling or cell cycle control, relapsed or refractory T-ALL carries a dismal survival rate below 30% [13,14,15,16]. This underscores the urgent need for novel molecular targets and less toxic therapeutic strategies.

### 1.2. The Non-Neuronal Cholinergic System in Leukemia

Accumulating evidence has demonstrated that lymphocytes possess an autonomous non-neuronal cholinergic system (NNCS) that regulates essential cellular processes, including growth, proliferation, differentiation, and immune responses [17,18,19]. This system comprises the biochemical machinery for acetylcholine (ACh) synthesis, mediated by choline acetyltransferase (ChAT), as well as its storage, release, and subsequent degradation by acetylcholinesterase (AChE), along with nicotinic and muscarinic acetylcholine receptors [17,20,21]. Importantly, components of this signaling network are overexpressed in multiple cancers, making them promising therapeutic targets [22,23,24]. Among these, the muscarinic M3 receptor subtype (mAChR M3) functions as a key regulator of cellular survival, triggering downstream pathways such as Mitogen-Activated Protein Kinase/Extracellular Signal-Regulated Kinase (MAPK/ERK) and Phosphoinositide 3-kinase/Protein Kinase B (PI3K/Akt) [25,26,27].

While mAChR M3 overexpression has been documented in several epithelial malignancies [28,29,30,31], its role in hematological tumors is highly relevant; it is functionally expressed in leukemic T-cell lines, such as Jurkat cells, where it regulates calcium signaling and cell survival [32,33]. Notably, mAChR M3 expression differs significantly between leukemic and normal hematopoietic cells [34]. Activation of mAChR M3 promotes resistance to apoptosis by upregulating anti-apoptotic proteins such as Bcl-2 [23,35], whereas pharmacological blockade of mAChR M3 inhibits tumor growth and enhances chemosensitivity [29,36,37]. Interestingly, cell survival, apoptosis resistance, and autophagy are also critically influenced by lysosomal proteases, highlighting a potential functional crosstalk between cholinergic signaling and proteolytic networks in cancer cells.

### 1.3. Lysosomal Cathepsins B and C in Malignant Survival

Cathepsins are lysosomal proteases involved in protein turnover, antigen processing, autophagy, and the regulation of cell death. In oncology, cathepsins B and C have attracted attention due to their roles in tumor progression, metastasis, immune modulation, and therapeutic resistance [38]. Under pathological conditions, these proteases can be secreted into the extracellular space, facilitating extracellular matrix remodeling and protumorigenic signaling networks [38,39,40]. Cathepsin C (dipeptidyl peptidase I) further contributes to tumor progression by regulating proteolytic and inflammatory pathways that support cancer cell growth and invasion [41]. In hematological malignancies, dysregulated lysosomal function and altered cathepsin activity are strongly implicated in leukemic cell survival, proliferation, and resistance to apoptosis [42]. Consequently, targeting cathepsins B and C represents a novel strategy to induce cancer cell death and enhance sensitivity to conventional therapies, offering a unique opportunity to bypass standard apoptotic resistance in T-ALL.

### 1.4. Darifenacin: Structural Versatility, Multi-Target Rationale

Darifenacin (DF) is a highly selective mAChR M3 antagonist approved for the treatment of overactive bladder syndrome [43,44]. Its high selectivity over other muscarinic subtypes confers a favorable safety profile, making it an attractive candidate for oncology drug repurposing [45]. Preclinical studies show that DF effectively inhibits tumor growth and reduces drug tolerance in solid tumor models [29,46].

Beyond its canonical affinity for the membrane-bound M3 receptor, DF possesses distinct structural and physicochemical properties. Specifically, the presence of a tertiary amine group on its pyrrolidine ring gives it the profile of a weakly basic, lipophilic amine, enabling it to diffuse across cell membranes and selectively accumulate via ion trapping within the acidic environment of lysosomes—the exact compartment where cathepsins B and C exert their proteolytic activity. This structural duality justifies a multi-target pharmacological approach: while its bulky substitutes drive surface receptor blockade, its overall profile facilitates cellular internalization and parallel interaction with lysosomal enzymes.

Achieving selective cytotoxicity in T-ALL is a notorious clinical challenge because leukemic cells share extensive biological similarities with normal T cells [47], a hurdle historically observed with agents such as arabinosylguanine [48]. However, the differential expression of mAChR M3 and altered lysosomal states establish a clear therapeutic window [49,50].

### 1.5. Objectives of the Study

Based on the aforementioned rationale, the present study systematically investigated the cytotoxic effects, cellular selectivity, and underlying molecular mechanisms of DF in the human T-ALL cell lines Jurkat and MOLT-4 compared to healthy human T lymphocytes.

Our primary aim was to evaluate its potential as a novel multi-target therapeutic strategy for T-ALL, determining whether its antileukemic efficacy relies on a dual pathway that simultaneously disrupts cholinergic signaling, induces glycative stress, inhibits lysosomal cathepsins, and triggers intrinsic mitochondrial apoptosis.

## 2. Results

### 2.1. Darifenacin Exhibits Selective Cytotoxicity Against Leukemic T Cells

**Figure 1:**
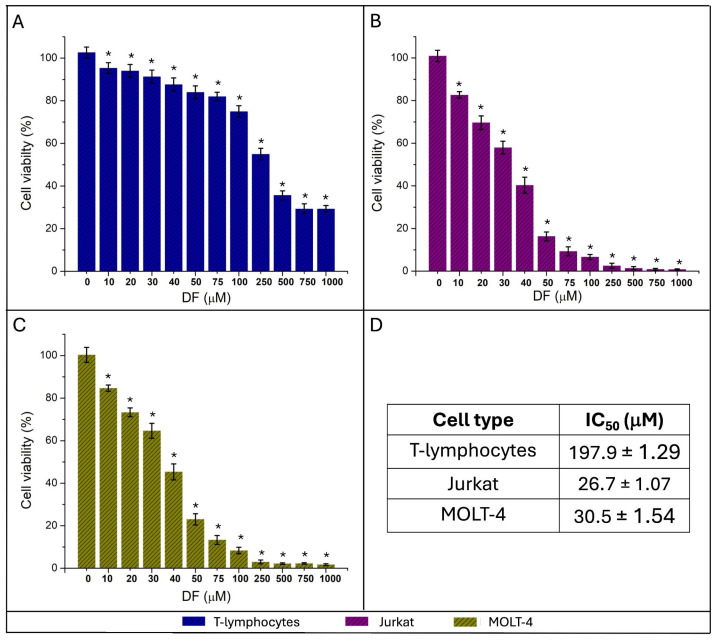
Determination of cell viability by MTT assays. Healthy T lymphocytes (**A**), Jurkat cells (**B**), and MOLT-4 cells (**C**) (1 × 10^5^ cells/well) were treated with increasing concentrations of DF (0, 10, 20, 30, 40, 50, 75, 100, 250, 500, 750, and 1000 µM) for 24 h. Cell viability was determined by the MTT assay and is expressed as the percentage of viable cells relative to the untreated control (100%). Data are presented as the mean ± Standard Deviation (SD) of three independent biological replicates. Statistical significance was determined using one-way Analysis of Variance (ANOVA) followed by Tukey’s multiple-comparison test, with *p* < 0.05 compared with the untreated control (*). (**D**) IC_50_ values obtained by nonlinear regression analysis of semi-logarithmic dose–response curves. The corresponding fitted dose–response curves (log_10_ DF concentration versus percentage cell viability) are presented in Supplementary Figure S1.

To evaluate the cytotoxic activity of DF and determine its selectivity toward leukemic cells, healthy phytohemagglutinin (PHA)-activated primary T lymphocytes and two T-ALL cell lines (Jurkat and MOLT-4) were exposed to increasing concentrations of DF (0–1000 µM) for 24 h, and cell viability was determined using the 3-(4,5-dimethylthiazol-2-yl)-2,5-diphenyltetrazolium bromide (MTT) assay (Figure 1).

As shown in Figure 1A, healthy T lymphocytes exhibited markedly greater resistance to DF-induced cytotoxicity than leukemic cells. Cell viability remained above 75% at concentrations up to 100 µM and gradually declined only at higher concentrations, yielding an apparent IC_50_ value of 197.9 ± 1.29 µM (Figure 1D). Although statistically significant reductions in viability relative to untreated controls were detected at 10 µM and above (*p* < 0.05), the magnitude of these changes remained modest across the concentration range that produced pronounced cytotoxicity in leukemic cells.

In contrast, both T-ALL cell lines displayed a marked concentration-dependent loss of viability (Figure 1B,C). Jurkat cells were particularly sensitive to DF, with viability decreasing to below 60% at 30 µM and below 20% at 50 µM, yielding an IC_50_ of 26.7 ± 1.07 µM (Figure 1D). Likewise, MOLT-4 cells exhibited a progressive reduction in viability across the concentration range tested, with an IC_50_ of 30.5 ± 1.54 µM, indicating only slightly lower sensitivity than Jurkat cells. In both leukemic cell lines, DF treatment produced statistically significant reductions in cell viability relative to untreated controls beginning at 10 µM (*p* < 0.05), with progressively greater effects observed as the drug concentration increased.

Comparison of IC_50_ values revealed a clear therapeutic window. DF was approximately 7.4-fold more potent against Jurkat cells and 6.5-fold more potent against MOLT-4 cells than against healthy T lymphocytes (Figure 1D). Moreover, concentrations between 20 and 50 µM produced substantial cytotoxicity in both leukemic cell lines while causing only minimal reductions in the viability of healthy T lymphocytes.

Semi-logarithmic dose–response curves generated by nonlinear regression (log DF concentration versus cell viability) and used to determine IC_50_ values are presented in Supplementary Figure S1.

Because the healthy T-lymphocyte response did not achieve complete inhibition within the tested concentration range, the corresponding IC_50_ should be regarded as an apparent value; nevertheless, the marked separation between the leukemic and normal cell dose–response curves clearly demonstrates the selective cytotoxic activity of DF toward T-ALL cells.

Consequently, these results demonstrate that DF exhibits a concentration-dependent, selective cytotoxicity against leukemic T cells while sparing healthy T lymphocytes, thereby establishing a favorable therapeutic window in vitro. This selective cytotoxic profile was independently confirmed by Trypan blue exclusion assays, which revealed that, while leukemic cell lines were highly responsive to DF, healthy activated T lymphocytes maintained robust viability even at 50 µM. Altogether, this distinct safety margin provides a solid basis that warrants further mechanistic investigation, as confirmed by the Trypan blue exclusion assays compiled in Supplementary Figure S2 and Supplementary Table S1.

### 2.2. Western Blot Analysis of mAChR M3, ChAT, and AChE in Healthy T Lymphocytes and Leukemic Cells

**Figure 2:**
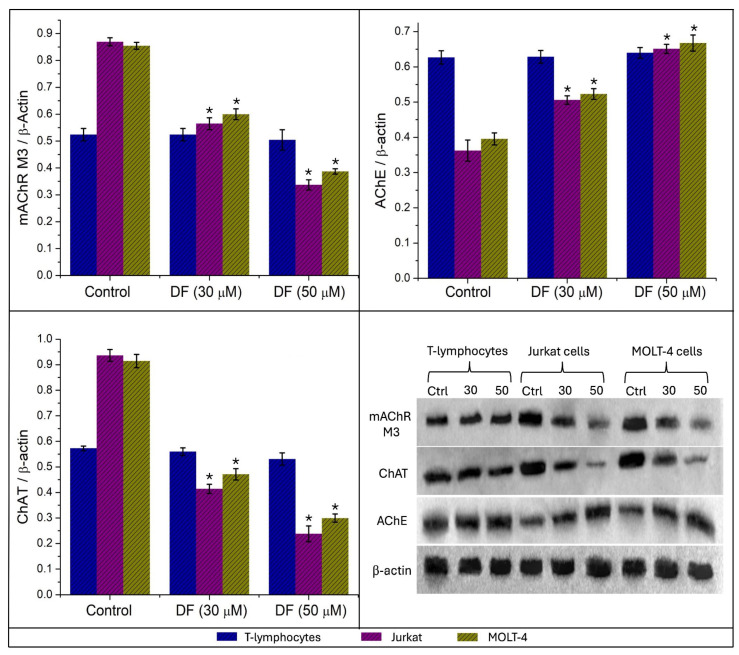
Western blot analysis of mAChR M3, ChAT, AChE, and β-actin loading control in healthy T lymphocytes, Jurkat cells, and MOLT-4 cells exposed to DF. Healthy T lymphocytes, Jurkat cells, and MOLT-4 cells were treated with increasing concentrations of DF (control 0, 30, and 50 µM) for 24 h at 37 °C. Following treatment, cells were lysed, and proteins were extracted using the RIPA method. A total of 100 µg of protein extract was loaded per lane. Primary antibodies against mAChR M3, ChAT, AChE, and β-Actin were used at a 1:1000 dilution in 0.1% TBS-T buffer supplemented with 1% BSA. Anti-mouse secondary antibody was used at a 1:3000 dilution in 0.1% TBS-T with 1% BSA. Representative blot images are shown alongside densitometric bar graphs (mean ± SD, *n* = 3) normalized to β-actin. mAChR M3 and ChAT expression decreased in a concentration-dependent manner in Jurkat and MOLT-4 cells, while AChE expression increased progressively. Healthy T lymphocytes showed no significant changes in any protein. Statistical analysis was performed using one-way ANOVA with Tukey–Kramer post hoc test; *p* ≤ 0.05. (*) indicates a statistically significant difference relative to the respective untreated control (0 µM DF). Full-length, uncropped blots for mAChR M3, ChAT, and AchE are provided in Supplementary Figure S3.

The expression of the NNCS components muscarinic acetylcholine receptor M3 (mAChR M3), choline acetyltransferase (ChAT), and acetylcholinesterase (AChE) was evaluated by Western blot in healthy T lymphocytes, Jurkat, and MOLT-4 cells following a 24 h treatment with DF (30 and 50 µM). Densitometric analyses, normalized to β-actin, are presented in Figure 2 (exact densitometric values are detailed in Supplementary Table S2).

Basal expression of mAChR M3 and ChAT was notably higher in leukemic cells than in healthy lymphocytes. Treatment with 50 µM DF profoundly downregulated these targets, reducing mAChR M3 expression by approximately 61% in Jurkat and 55% in MOLT-4 cells. Similarly, ChAT expression decreased by 75% and 67% in Jurkat and MOLT-4 cells, respectively, relative to untreated controls.

Conversely, basal AChE expression was initially lower in leukemic cells compared to healthy lymphocytes but increased significantly upon DF exposure. At 50 µM DF, AChE levels rose by approximately 80% in Jurkat and 69% in MOLT-4 cells.

Collectively, these results demonstrate that DF selectively modulates the expression of NNCS components in leukemic cells without significantly affecting healthy T lymphocytes. The concomitant downregulation of mAChR M3 and ChAT, alongside the marked upregulation of AChE, suggests a robust disruption in cholinergic signaling specific to malignant cells. Statistical significance was assessed using one-way ANOVA with Tukey–Kramer post hoc test (*p* ≤ 0.05), as shown in Figure 2.

### 2.3. Darifenacin Selectively Triggers Programmed Cell Death via Apoptosis in Leukemic T Cells

**Figure 3:**
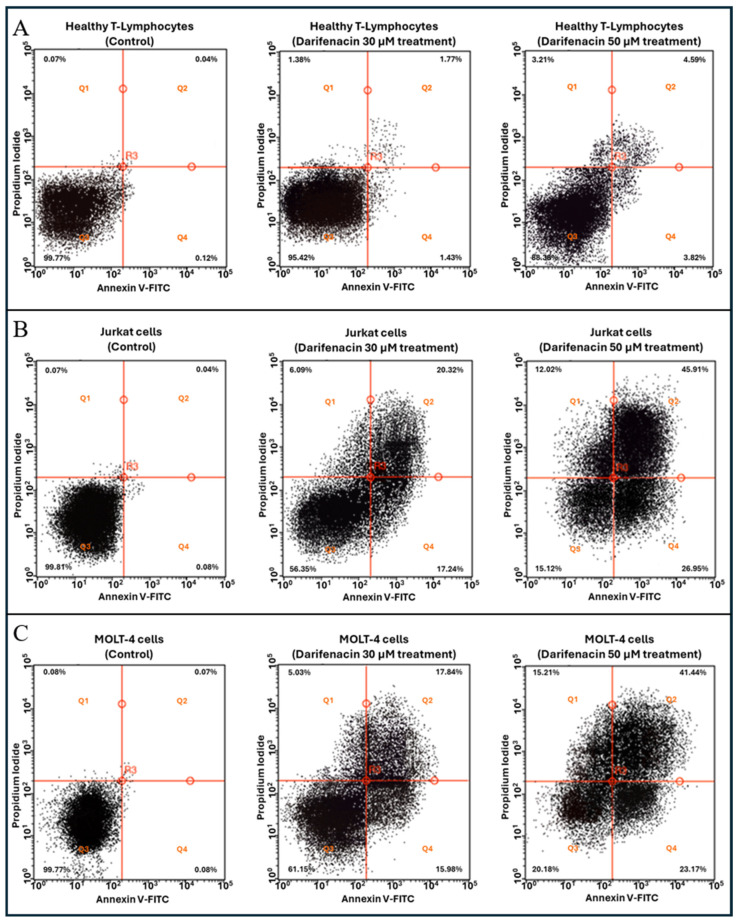
Flow cytometric analysis of apoptosis following DF treatment. (**A**) Healthy T lymphocytes, (**B**) Jurkat cells, and (**C**) MOLT-4 cells were treated with increased concentrations of DF (control 0, 30, and 50 µM) for 24 h at 37 °C. After incubation, cells were harvested, washed, and resuspended at a density of 1 × 10^6^ cells/mL. Subsequently, they were stained with annexin V-FITC and PI to distinguish viable cells from those undergoing early or late apoptosis or necrosis. Flow cytometry analysis was performed by acquiring 30,000 events per sample within the morphologically gated population. Quadrants are defined as Q1: necrotic cells (annexin V^−^/PI^+^); Q2: late apoptotic cells (annexin V^+^/PI^+^); Q3: viable cells (annexin V^−^/PI^−^); and Q4: early apoptotic cells (annexin V^+^/PI^−^). Representative dot plots are shown from three independent biological experiments (*n* = 3); plots corresponding to the remaining two independent replicates are provided in Supplementary Figure S5. Quantitative data summarizing the cell populations are presented as mean ± SD in Supplementary Table S3.

To determine whether the DF-induced reduction in cell viability was due to the activation of regulated cell death pathways rather than non-specific necrosis, an Annexin V-Fluorescein Isothiocyanate (FITC)/Propidium Iodide (PI) double-staining assay was performed by flow cytometry (*n* = 3). The baseline viability of all untreated controls exceeded 99.7% (Figure 3 and Supplementary Table S3), validating the integrity of the experimental setup and the absence of vehicle-induced artifacts (Supplementary Figure S4).

Treatment with DF induced a severe, concentration-dependent shift toward apoptosis in both malignant T cell lines after 24 h (Figure 3B,C). In Jurkat cells, exposure to 30 µM of DF reduced viability to 57.78% ± 1.38%, with a concurrent increase in total apoptotic cells (early + late apoptosis) to 36.48%. At the highest concentration tested (50 µM), the cytotoxic impact was stark: viable Jurkat cells plummeted to 14.84% ± 0.34%, driven by a massive induction of apoptosis, which reached 73.45% (27.13% ± 0.22% early apoptotic and 46.32% ± 0.47% late apoptotic populations; Figure 3B). Conversely, mechanical necrosis remained minimal at 11.71% ± 0.49%.

A highly consistent pro-apoptotic profile was observed in MOLT-4 cells (Figure 3C). Treatment with 30 µM of DF reduced viability to 61.84% ± 0.69% (33.04% total apoptosis). Upon increasing the concentration to 50 µM, the viable cell pool fell to 20.80% ± 0.55%, while the total apoptotic population rose significantly to 64.58% (23.30% ± 0.55% early apoptotic and 41.28% ± 0.78% late apoptotic cells), with only 14.62% ± 0.63% registering as necrotic.

Crucially, under identical experimental and proliferative conditions, healthy PHA-activated primary T lymphocytes exhibited remarkable resilience to DF-mediated programmed cell death (Figure 3A). Exposure to 30 µM of DF resulted in a negligible change in viability (95.67% ± 0.24%). Even at the maximum concentration of 50 µM, 88.42% ± 1.43% of the healthy T lymphocytes remained completely viable and double-negative (Annexin V^−^/PI^−^), showing marginal apoptotic (8.26%) or necrotic (3.32% ± 0.46%) signals. These quantitative findings, as shown in Figure 3 and summarized in Supplementary Table S3, confirm that DF selectively induces apoptotic cell death in leukemic targets while sparing healthy immune counterparts. Additional representative cytometric replicates are detailed in Supplementary Figure S5.

### 2.4. Darifenacin Induces the Accumulation of MG and AGEs in T-ALL Cells

**Figure 4:**
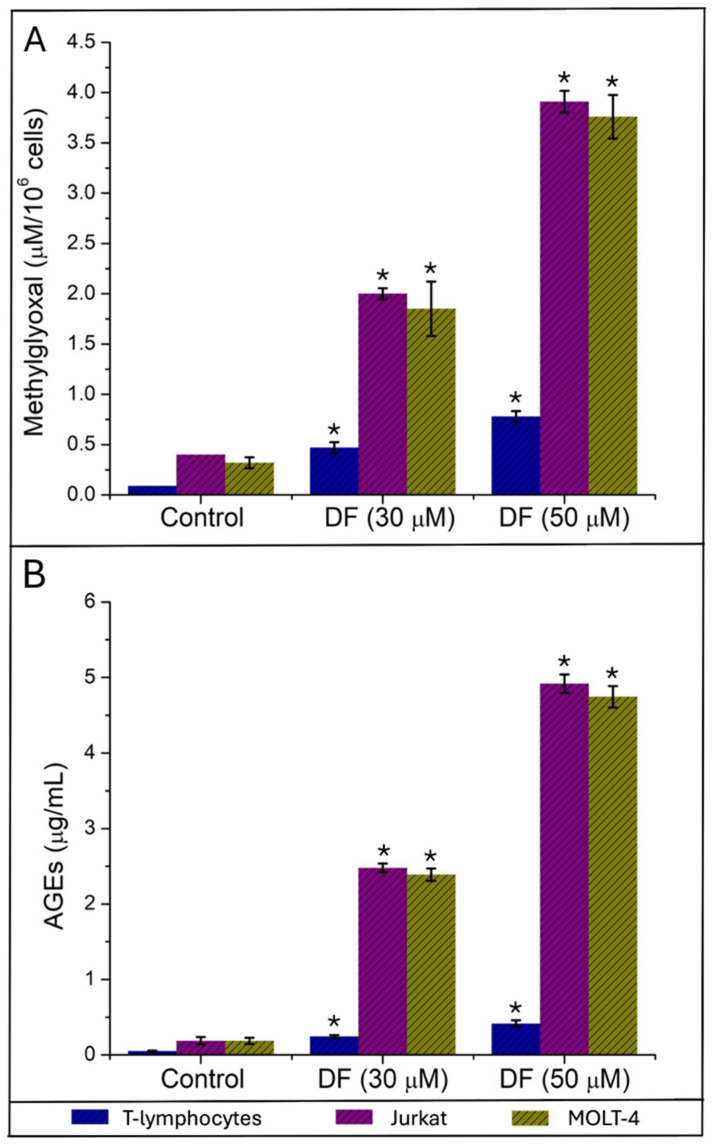
Determination of intracellular MG and AGE levels following DF treatment. Healthy T lymphocytes, Jurkat cells, and MOLT-4 cells were treated with increasing concentrations of DF (control 0, 30, and 50 µM) for 24 h at 37 °C. After treatment, 1 × 10^6^ cells per condition were harvested, washed with cold PBS, and lysed by five freeze–thaw cycles in liquid nitrogen and at 37 °C. (**A**) Intracellular MG levels were determined by a colorimetric assay based on the reaction of MG with 2,4-dinitrophenylhydrazine (DNPH), measured at 432 nm, and expressed as µM MG per 1 × 10^6^ cells. (**B**) AGEs were quantified by Enzyme-Linked Immunosorbent Assay (ELISA) (MyBioSource, San Diego, CA, USA) following 1:100 dilution of protein-normalized lysates (1 mg/mL), with absorbance measured at 450 nm; results are expressed as µg AGEs per mL of cell lysate. Data represent the mean ± standard deviation of three independent biological replicates. Statistical analysis was performed using one-way ANOVA, followed by Tukey’s post hoc test; *p* < 0.05. (*) indicates a statistically significant difference relative to the respective untreated control (0 µM DF).

To evaluate the effect of DF on intracellular glycative stress, levels of MG and AGEs were quantified in healthy T lymphocytes, Jurkat cells, and MOLT-4 cells after treatment with DF at increasing concentrations (control [0], 30, and 50 µM). Results are expressed as µM MG per 1 × 10^6^ cells and as µg AGEs per mL of cell lysate, respectively, and are presented in Figure 4.

Regarding MG accumulation, healthy T lymphocytes exhibited low basal MG levels (0.092 µM), which underwent a modest but progressive increase upon DF treatment, reaching 0.473 ± 0.108 µM at 30 µM and 0.779 ± 0.108 µM at 50 µM (~5- and 8.5-fold increases relative to the untreated control, respectively). In contrast, both leukemic cell lines demonstrated a pronounced, concentration-dependent surge in this reactive carbonyl species (*p* < 0.05). In Jurkat cells, baseline MG levels (0.397 ± 0.000 µM) increased to 2.000 ± 0.108 µM at 30 µM DF and to 3.908 ± 0.216 µM at 50 µM DF, representing approximately 5- and 9.8-fold increases, respectively. Similarly, MOLT-4 cells showed a robust increase from a baseline of 0.321 ± 0.108 µM to 1.847 ± 0.540 µM and 3.756 ± 0.432 µM under 30 and 50 µM DF conditions, respectively, corresponding to up to an 11.7-fold increase over untreated cells. Across all models, the leukemic lines accumulated substantially higher absolute amounts of MG than their non-malignant counterparts.

A parallel, even more pronounced trend was observed in AGE accumulation. Baseline AGEs levels were minimal in healthy T lymphocytes (0.051 ± 0.005 µg/mL) and increased gradually to 0.244 ± 0.021 µg/mL and 0.414 ± 0.046 µg/mL following treatments with 30 and 50 µM DF, respectively. Conversely, leukemic cells displayed a striking vulnerability to DF-induced advanced glycation. In Jurkat cells, basal AGEs levels (0.186 ± 0.051 µg/mL) increased sharply to 2.479 ± 0.056 µg/mL at 30 µM and 4.917 ± 0.123 µg/mL at 50 µM, representing substantial 13.4- and 26.5-fold increases over controls (*p* < 0.05). This profile was closely mirrored by MOLT-4 cells, in which baseline values (0.186 ± 0.041 µg/mL) increased to 2.388 ± 0.082 µg/mL and 4.744 ± 0.144 µg/mL at 30 and 50 µM DF, respectively, representing approximate 12.8- and 25.5-fold increases.

Taken together, these findings demonstrate that DF treatment triggers a significant, concentration-dependent accumulation of both MG and AGEs. This effect was profoundly more prominent in leukemic cell lines than in healthy T lymphocytes, strongly suggesting that DF selectively promotes glycative stress within the tumor microenvironment. The synchronous rise of MG, a highly reactive dicarbonyl precursor, and the formation of downstream AGEs underscore activation of a potent glycative stress pathway that likely drives the cytotoxic and apoptotic mechanisms observed in T-ALL cells.

### 2.5. Darifenacin Activates Caspase-3 and Modulates Bcl-2 Family Proteins in Leukemic Cells

**Figure 5:**
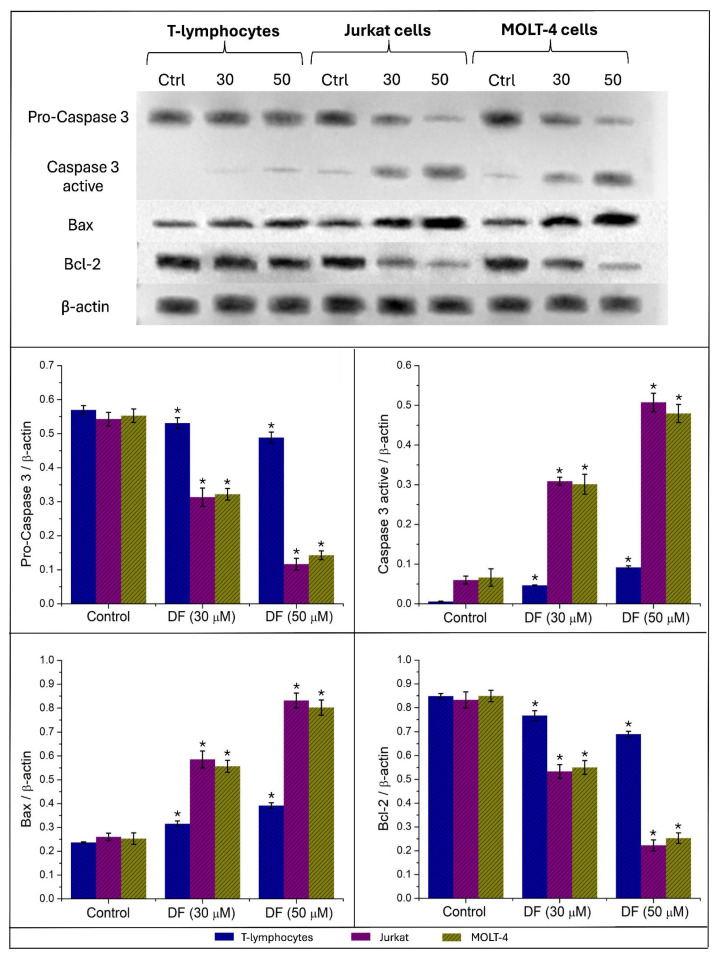
Western blot analysis of Pro-caspase 3/Caspase 3 active, Bax, Bcl-2, and β-actin loading control in healthy T lymphocytes, Jurkat cells, and MOLT-4 cells exposed to DF. Healthy T lymphocytes, Jurkat cells, and MOLT-4 cells were treated with increasing concentrations of DF (control 0, 30, and 50 µM) for 24 h at 37 °C. Following treatment, cells were lysed, and proteins were extracted using the RIPA method. A total of 100 µg of protein extract was loaded per lane. Primary antibodies against Pro-caspase 3/Caspase 3 active, Bax, Bcl-2, and β-Actin were used at a 1:1000 dilution in 0.1% TBS-T buffer supplemented with 1% BSA. Anti-mouse secondary antibody was used at a 1:3000 dilution in 0.1% TBS-T with 1% BSA. Representative blot images are shown alongside densitometric bar graphs (mean ± SD, *n* = 3) normalized to β-actin. Statistical analysis was performed using one-way ANOVA with Tukey–Kramer post hoc test; *p* ≤ 0.05. (*) indicates a statistically significant difference relative to the respective untreated control (0 µM DF). Full-length, uncropped blots of caspase-3, Bax, and Bcl-2 are provided in Supplementary Figure S6.

To further characterize the molecular mechanisms of DF-induced apoptosis, we examined caspase-3 activation and the expression of the mitochondrial Bcl-2 family proteins (Bax and Bcl-2). Expression levels following a 24 h treatment with DF (30 and 50 µM) were assessed by Western blot in healthy T lymphocytes, Jurkat, and MOLT-4 cells. Densitometric analyses are presented in Figure 5 (exact values for all conditions are detailed in Supplementary Table S4).

Caspase-3 activation. In Jurkat cells, pro-caspase-3 levels decreased markedly in a concentration-dependent manner, accompanied by a robust increase in active caspase-3 upon treatment with 30 µM and 50 µM DF. A similar pro-apoptotic trend was observed in MOLT-4 cells, where pro-caspase-3 levels declined significantly, and active caspase-3 rose sharply at the higher concentration of 50 µM. In contrast, healthy T lymphocytes exhibited only a minimal, non-significant increase in active caspase-3 even at 50 µM DF, with pro-caspase-3 remaining largely unchanged.

Bax and Bcl-2 expression. DF treatment induced a profound, dose-dependent shift in the balance of Bcl-2 family proteins in both leukemic lines. Bax expression increased substantially in Jurkat and MOLT-4 cells after exposure to 30 µM and 50 µM DF. Concurrently, anti-apoptotic Bcl-2 expression declined precipitously in both malignant lines. These reciprocal changes led to a dramatic increase in the Bax/Bcl-2 ratio at 50 µM DF, rising more than 10-fold in both Jurkat (from 0.312 to 3.736) and MOLT-4 cells (from 0.298 to 3.168). In healthy T lymphocytes, however, the increase in Bax was modest, and Bcl-2 levels remained elevated, resulting in only a minor shift in the Bax/Bcl-2 ratio (reaching 0.568 at 50 µM).

These results strongly suggest that DF selectively activates the intrinsic mitochondrial apoptotic pathway in leukemic T cells, but not in healthy T lymphocytes, by upregulating Bax, downregulating Bcl-2, and triggering the caspase-3 executioner cascade. Statistical significance was assessed using one-way ANOVA with Tukey–Kramer post hoc testing (*p* ≤ 0.05), as shown in Figure 5.

### 2.6. Cathepsin B Activity and Inhibition Profiles

We examined the dose-dependent inhibitory effects of DF on Cathepsin B activity across three distinct cell populations: healthy T lymphocytes, Jurkat cells, and MOLT-4 cells. Cathepsin B activity was quantified by measuring the fluorescence slope (arbitrary units per minute, A.U./min) and subsequently normalized to residual activity as a percentage relative to untreated controls.

**Figure 6:**
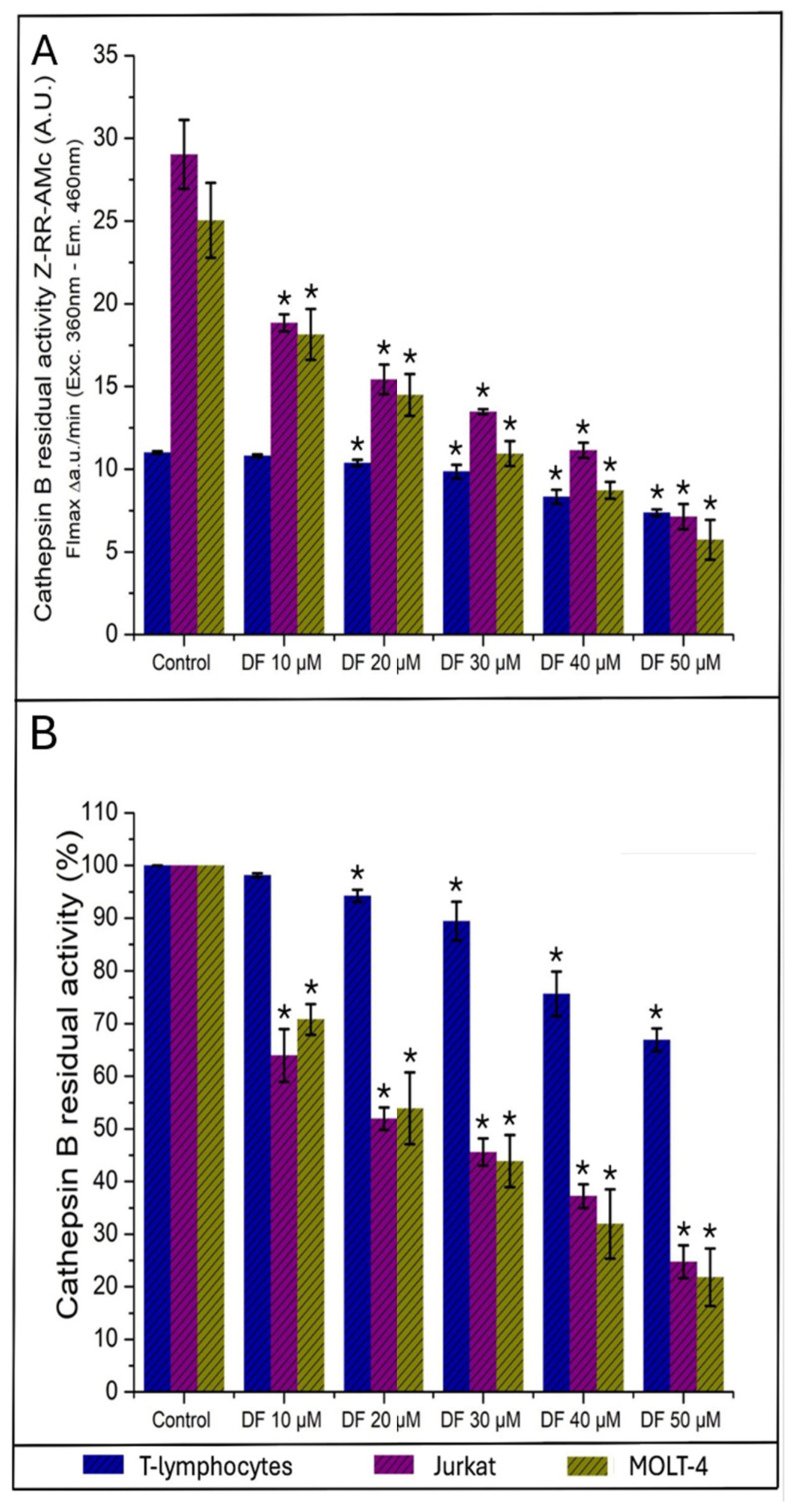
Cathepsin B activity in Healthy T lymphocytes, Jurkat cells, and MOLT-4 cells. (**A**) Cathepsin B activity measured as fluorescence slope (IF A.U./min) in healthy T lymphocytes, Jurkat cells, and MOLT-4 cells treated with increasing concentrations of DF (control 0, 30, and 50 µM) for 24 h at 37 °C. (**B**) Cathepsin B residual activity (%) in healthy T lymphocytes, Jurkat cells, and MOLT-4 cells following treatment with DF at concentrations ranging from 10 to 50 µM. Control groups (0 µM) were normalized to 100% activity. Data correspond to three independent biological replicates and are presented as the mean, with bars indicating experimental standard deviation. Statistical analysis was performed using one-way ANOVA followed by Tukey’s post hoc test, with statistical significance defined as *p* < 0.05 (* with respect to the corresponding control).

As illustrated in Figure 6A, baseline Cathepsin B activity varied substantially among the three cell types, with Jurkat cells exhibiting the highest absolute activity (29.17 ± 2.53 IF A.U./min), followed by MOLT-4 cells (25.00 ± 2.08 IF A.U./min), and healthy T lymphocytes (11.00 ± 0.17 IF A.U./min). The application of DF induced concentration-dependent reductions in enzymatic activity across all cell types, with the magnitude of inhibition displaying marked cell-type specificity.

The normalized residual activity data presented in Figure 6B reveal distinct inhibition profiles. Healthy T lymphocytes exhibited remarkable resistance to DF-mediated inhibition, maintaining 98.15 ± 0.37% residual activity at 10 µM DF and retaining 66.92 ± 2.14% activity even at the highest concentration tested (50 µM). In stark contrast, both leukemic cell lines exhibited substantially greater sensitivity to DF treatment. Jurkat cells showed progressive inhibition from 63.96 ± 5.02% at 10 µM to 24.78 ± 3.13% at 50 µM, representing a 75.22% reduction in enzymatic activity. Similarly, MOLT-4 cells displayed residual activity that declined from 70.80 ± 2.93% at 10 µM to 21.83 ± 5.46% at 50 µM, corresponding to 78.17% inhibition at the maximum concentration tested.

The dose–response relationships for Cathepsin B inhibition were nearly linear in leukemic cells, with correlation coefficients indicating consistent inhibitory potency across the concentration range. The calculated IC_50_ values (concentrations producing 50% inhibition) were 22.3 ± 1.1 µM for Jurkat cells and 19.8 ± 1.4 µM for MOLT-4 cells. In contrast, healthy T lymphocytes did not reach 50% inhibition within the tested concentration range, indicating an IC_50_ value exceeding 50 µM.

### 2.7. Cathepsin C Activity and Inhibition Profiles

Cathepsin C activity was assessed using spectrophotometric-based enzymatic assays, with A.U. serving as the primary activity metric. The baseline activity levels and inhibition patterns of Cathepsin C diverged substantially from those of Cathepsin B, revealing enzyme-specific responses to DF treatment.

**Figure 7:**
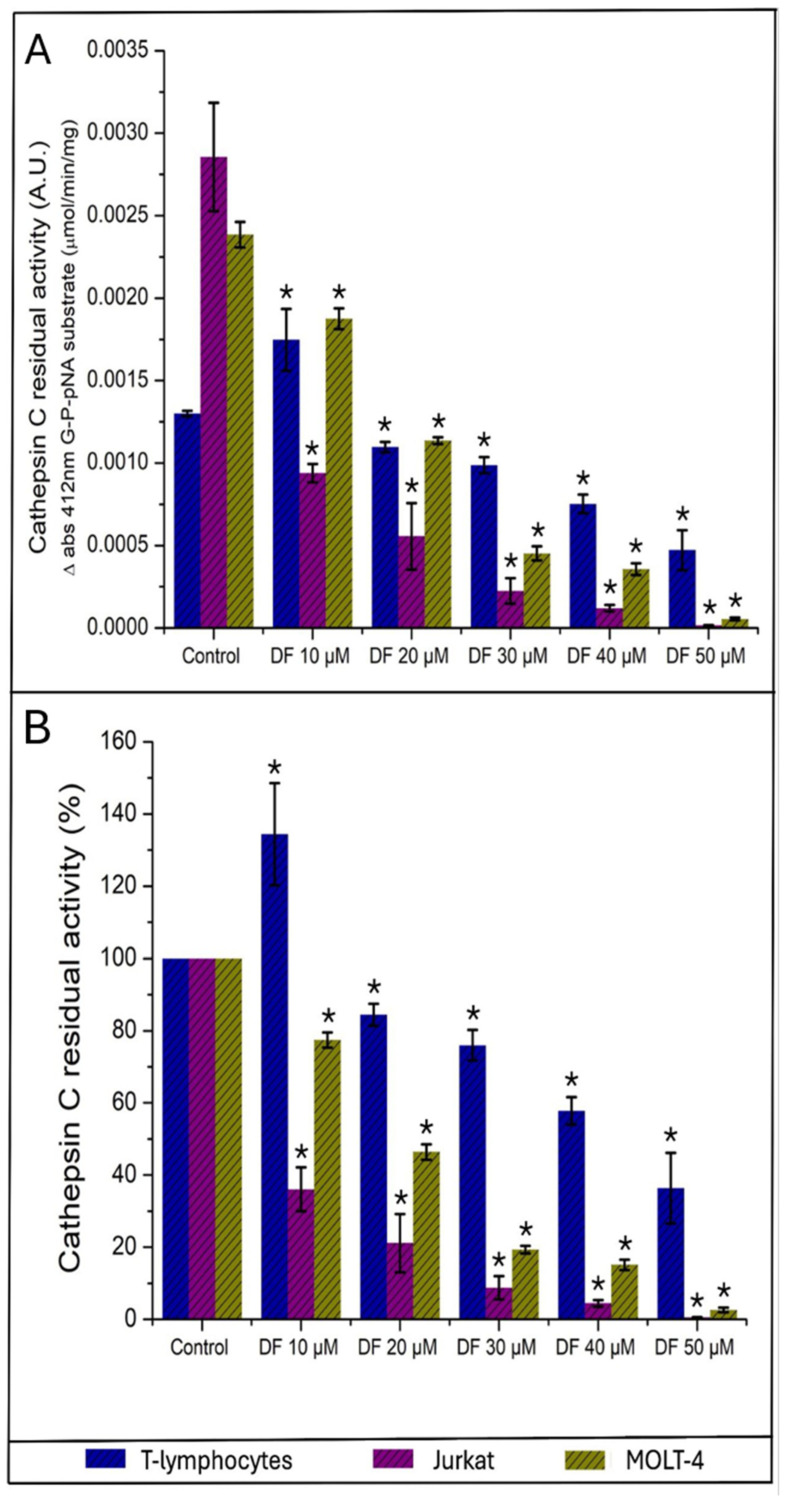
Cathepsin C activity in healthy T lymphocytes, Jurkat cells, and MOLT-4 cells. (**A**) Cathepsin C activity measured in absorbance units (×10^−3^ A.U.) at 412 nm in healthy T lymphocytes, Jurkat cells, and MOLT-4 cells exposed to increasing concentrations of DF (control 0, 30, and 50 µM) for 24 h at 37 °C. (**B**) Cathepsin C residual activity (%) across the three cell types following DF treatment. Control conditions (0 µM) were set for 100% activity. Data correspond to three independent biological replicates and are presented as the mean, with bars indicating experimental standard deviation. Statistical analysis was performed using one-way ANOVA followed by Tukey’s post hoc test, with statistical significance defined as *p* < 0.05 (* with respect to the corresponding control).

Figure 7A demonstrates that Jurkat cells exhibited the highest basal Cathepsin C activity (2.88 ± 0.31 × 10^−3^ A.U.), followed by MOLT-4 cells (2.38 ± 0.08 × 10^−3^ A.U.) and healthy T lymphocytes (1.30 ± 0.04 × 10^−3^ A.U.). A notable and unexpected observation emerged at the 10 µM DF concentration in healthy T lymphocytes, where Cathepsin C activity increased to 1.75 ± 0.18 ×10^−3^ units, representing a 34.35% increase over control levels. This paradoxical activation was not observed in either leukemic cell line and warrants further mechanistic investigation.

The residual activity analysis presented in Figure 7B reveals that Cathepsin C in leukemic cells exhibited extraordinary sensitivity to DF inhibition, substantially exceeding that observed for Cathepsin B. Jurkat cells demonstrated the most pronounced inhibition profile, with residual activity plummeting from 36.04 ± 6.06% at 10 µM to near-complete ablation at 50 µM (0.57 ± 0.03%), representing a 99.43% inhibition. MOLT-4 cells similarly displayed robust inhibition, declining from 77.41 ± 2.09% at 10 µM to 2.60 ± 0.63% at 50 µM (97.40% inhibition).

The IC_50_ values for Cathepsin C inhibition in leukemic cells were markedly lower than those for Cathepsin B. Jurkat cells exhibited an estimated IC_50_ of 8.4 ± 0.7 µM, while MOLT-4 cells demonstrated an IC_50_ of 16.2 ± 0.9 µM. In contrast, healthy T lymphocytes, despite the initial activation at 10 µM, showed progressive inhibition at higher concentrations, with residual activity declining to 36.36 ± 9.77% at 50 µM, yielding an estimated IC_50_ of 46.8 ± 2.3 µM.

Making a quantitative comparison of the inhibition by DF to 30 µM, we observed that the healthy T lymphocytes retained 89.46 ± 3.67% Cathepsin B activity and 75.97 ± 4.19% Cathepsin C activity, whereas Jurkat cells retained only 45.58 ± 2.56% and 8.73 ± 3.24%, respectively, and MOLT-4 cells retained 43.87 ± 4.94% and 19.31 ± 1.06%, respectively. This concentration represents a potential therapeutic window in which leukemic cells exhibit substantial inhibition of cathepsin activity while healthy T cells maintain near-normal enzymatic function.

### 2.8. Molecular Docking Studies

To generate mechanistic hypotheses regarding the inhibitory effects of DF on the studied cathepsins, in silico molecular docking analyses were performed using the crystallographic structures of human cathepsin B (PDB ID: 8B4T) [51] and human cathepsin C (PDB ID: 6IC7) [52], retrieved from the Protein Data Bank (PDB) [53]. Protein structures were prepared by ligand removal, protonation, and energy minimization prior to docking simulations. Docking calculations were performed using the CB-Dock server under standard conditions [54].

**Figure 8:**
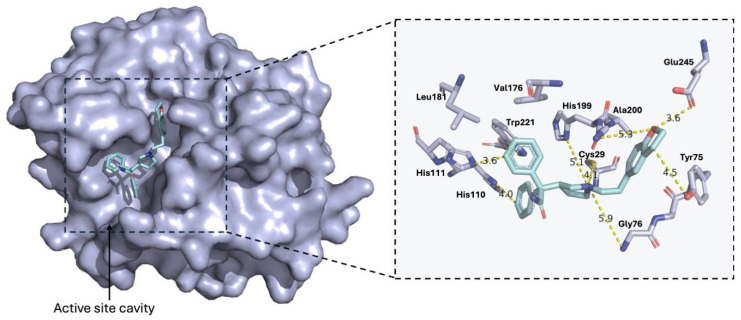
Molecular docking of DF into the catalytic pocket of human cathepsin B (PDB ID: 8B4T). Left, surface representation of cathepsin B showing DF bound within the catalytic cavity. Right, enlarged view of the docked complex illustrating the principal molecular interactions established between DF and active-site residues. DF is shown in cyan stick representation, while residues involved in ligand recognition and stabilization are highlighted around the binding pocket. The diphenyl groups form hydrophobic and π-stacking interactions with Trp221 and His110/His111, whereas the pyrrolidine ring is oriented toward His199 and the catalytic residue Cys29. In addition, the dihydrobenzofuran moiety establishes polar and aromatic contacts with Glu245 and Tyr75, contributing to stabilization of the ligand within the catalytic site. Distances are indicated in angstroms (Å). Molecular representations were generated using PyMOL Molecular Graphics System v2.5.0 (Schrödinger, LLC, New York, NY, USA).

Docking simulations revealed that DF docked efficiently within the substrate-binding cleft of Cathepsin B, exhibiting a predicted binding affinity of −8.6 kcal/mol, which was more favorable than the affinity calculated for the co-crystallized reference inhibitor (Inhibitor 7, −6.3 kcal/mol) (Supplementary Figure S7). The predicted stability of the complex was supported by multiple coordinated non-covalent interactions involving hydrophobic, aromatic, and polar residues associated with substrate recognition and catalytic stabilization (Figure 8). The two diphenyl moieties of DF were positioned within a predominantly hydrophobic region formed by Trp221, His110, and His111, maintaining average interaction distances of approximately 3.7 Å. These contacts are consistent with stabilizing hydrophobic and π-π stacking interactions within the occluding loop region of Cathepsin B. In addition, the pyrrolidine ring was oriented toward the catalytic core, where the N1 atom was located 5.1 Å from the ND1 atom of His199, suggesting possible electrostatic or long-range polar interactions within the catalytic microenvironment. Notably, the catalytic residue Cys29 was positioned 4.1 Å from the N1 atom of the pyrrolidine ring, indicating close spatial proximity between DF and the catalytic center of the enzyme, potentially contributing to active-site occupancy and inhibitory stabilization.

At the opposite end of the molecule, the dihydrobenzofuran moiety established additional stabilizing interactions. The O2 atom interacted with the OE2 atom of Glu245 at approximately 3.5 Å, compatible with a directed polar interaction or hydrogen bond. Simultaneously, the aromatic benzofuran ring maintained a π-stacking interaction with Tyr75 at approximately 3.7 Å. Altogether, these interactions suggest that DF simultaneously occupies hydrophobic and polar microenvironments within the catalytic cleft of Cathepsin B, promoting formation of a favorable ligand-protein complex.

Similarly, docking simulations demonstrated that DF binds favorably within the catalytic cavity of Cathepsin C, displaying a predicted binding affinity of −8.1 kcal/mol. This affinity was higher than the calculated value for the co-crystallized inhibitory ligand in the 6IC7 structure (−7.5 kcal/mol), supporting the formation of a favorable interaction within the active-site environment (Supplementary Figure S8).

**Figure 9:**
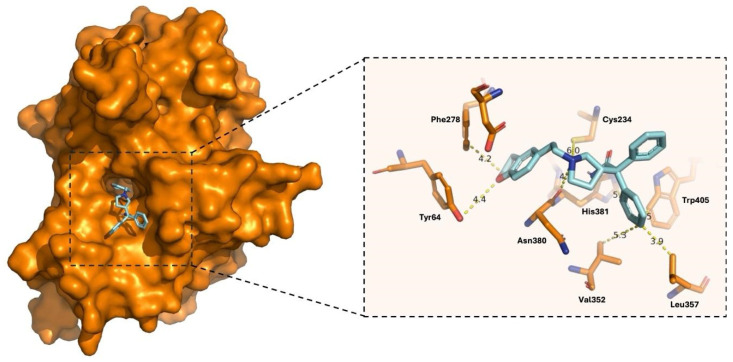
Molecular docking of DF into the catalytic cavity of human cathepsin C (PDB ID: 6IC7). Left, surface representation of cathepsin C showing DF bound within the catalytic pocket. Right, enlarged view of the docked complex illustrating the principal molecular interactions established between DF and residues lining the active site. DF is shown in cyan stick representation, while residues involved in ligand recognition and catalytic stabilization are highlighted around the binding cavity. The diphenyl groups establish hydrophobic and aromatic interactions with Trp405, Leu357, and Val352, whereas the pyrrolidine ring is positioned toward the catalytic region in proximity to the catalytic residue Cys234, as well as His381 and Asn380. In addition, the dihydrobenzofuran moiety forms polar and aromatic contacts with Tyr64 and Phe278, contributing to stabilization of the ligand within the catalytic site. Distances are indicated in angstroms (Å). Molecular representations were generated using PyMOL Molecular Graphics System v2.5.0 (Schrödinger, LLC, New York, NY, USA).

The predicted binding mode in Cathepsin C involved multiple hydrophobic, aromatic, and polar contacts distributed throughout the catalytic cleft (Figure 9). The diphenyl groups of DF were oriented toward a hydrophobic region composed of Leu357, Val352, and Trp405, maintaining interaction distances of approximately 4.5, 5.3, and 3.9 Å, respectively. These contacts are compatible with hydrophobic packing and aromatic stabilization within the substrate-recognition pocket, while Trp405 likely contributes to additional π-π interactions with the ligand’s phenyl rings.

The pyrrolidine ring was positioned near the enzyme’s catalytic region. The N1 atom was located 6.0 Å from the sulfur atom (SG) of the catalytic residue Cys234, 5.0 Å from His381, and 4.2 Å from the carbonyl oxygen of Asn380. These distances suggest that the tertiary amine of DF is oriented toward the catalytic microenvironment, potentially enabling long-range electrostatic stabilization and polar interactions with residues involved in substrate processing and catalysis. The proximity of the ligand to the catalytic Cys further supports effective occupation of the active-site cavity.

Additional stabilization was observed through the dihydrobenzofuran moiety. The O2 atom of this group was positioned 4.4 Å from the hydroxyl group of Tyr64 and 4.2 Å from the CE2 atom of Phe278, indicating combined polar and aromatic interactions that may contribute to ligand anchoring and conformational stabilization within the catalytic cleft. These observations suggest that the aromatic and heterocyclic regions of DF adapt efficiently to both hydrophobic and partially polar regions of the Cathepsin C active site.

## 3. Discussion

The present study provides the first evidence that DF exerts targeted antileukemic activity against T-ALL cells, while demonstrating minimal effects against normal T lymphocytes. In general, our findings suggest that DF induces apoptosis through mechanisms associated with glycative stress and activation of the intrinsic mitochondrial pathway, as evidenced by increased Bax/Bcl-2 ratios and caspase-3 activation.

### 3.1. Differential Expression of Non-Neuronal Cholinergic System Components Underlies Selective Cytotoxicity

The selective antileukemic activity of DF appears to be associated with differential expression of NNCS components between leukemic and normal T cells. Specifically, leukemic cells exhibited increased expression of mAChR M3 and ChAT, together with reduced AChE levels, a profile consistent with enhanced autocrine cholinergic signaling previously reported in T-ALL [23]. This altered cholinergic phenotype may provide a therapeutic window for selective targeting.

Indeed, DF displayed marked selective cytotoxicity, with IC_50_ values of 26.7 ± 1.07 µM and 30.5 ± 1.54 µM in Jurkat and MOLT-4 cells, respectively, whereas healthy T lymphocytes remained largely unaffected at comparable concentrations [43]. This degree of selectivity is comparable to that reported for other T-cell-selective agents [49,50] and likely reflects differences in receptor signaling, metabolic vulnerabilities, and apoptotic thresholds between malignant and healthy T cells. Our findings extend previous reports of the anticancer activity of DF in solid tumors to hematological malignancies. DF has been shown to inhibit tumor growth and survival in colorectal and gastric cancer models by blocking mAChR M3 signaling [28,29]. Together with the high selectivity of DF for the M3 receptor subtype [45], these observations support the therapeutic potential of targeting cholinergic signaling pathways in T-ALL.

### 3.2. Glycative Stress as a Mechanism of Darifenacin-Induced Antileukemic Activity

A particularly notable finding of this study was the marked accumulation of MG and AGEs in DF-treated leukemic cells, with minimal effects in healthy T lymphocytes. This observation suggests that DF exploits a leukemia-specific metabolic vulnerability by inducing glycative stress. MG, a highly reactive glycolytic byproduct, exerts dose-dependent effects in cancer, promoting tumor growth at low concentrations while inducing cytotoxicity at high levels [55]. Our findings are consistent with the latter mechanism, whereby DF-induced MG accumulation reaches toxic levels in leukemic cells.

The mechanisms linking M3 receptor blockade to MG accumulation remain to be elucidated. Because M3 signaling regulates intracellular calcium homeostasis [32,35], which in turn influences metabolic pathways and carbonyl stress responses, disruption of this signaling axis may impair cellular defenses against MG accumulation. In this context, glyoxalase 1 (GLO1), the principal enzyme responsible for MG detoxification and a recognized metabolic vulnerability in cancer cells, represents a plausible target of DF-mediated effects [56]. Although GLO1 activity was not evaluated in this study, the substantial increase in MG levels suggests that DF disrupts pathways involved in MG homeostasis.

Importantly, MG and AGEs promote oxidative stress and mitochondrial dysfunction, leading to activation of the intrinsic apoptotic pathway [57]. Previous studies have shown that MG-derived AGEs increase the Bax/Bcl-2 ratio and activate caspase-dependent apoptosis through mitochondrial signaling [58,59]. Accordingly, the marked elevation of the Bax/Bcl-2 ratio and robust caspase-3 activation observed following DF treatment strongly support a glycative stress-mediated apoptotic mechanism in T-ALL cells.

### 3.3. Darifenacin Induces Apoptosis Through the Intrinsic Pathway

Our results demonstrate that DF induces apoptosis predominantly through activation of the intrinsic mitochondrial pathway, as evidenced by the marked increase in the Bax/Bcl-2 ratio and robust caspase-3 activation in both leukemic cell lines. The pronounced shift toward a pro-apoptotic phenotype observed following DF treatment is consistent with the activation of mitochondrial apoptosis reported for other antileukemic agents [60].

The dose-dependent activation of caspase-3 further confirms the engagement of the apoptotic machinery. Notably, healthy T lymphocytes exhibited only minimal caspase-3 activation, supporting the selective cytotoxicity of DF. This selectivity is particularly relevant, as conventional chemotherapeutic agents often induce substantial apoptosis in healthy T lymphocytes, contributing to immunosuppression and treatment-related toxicity.

Targeting the intrinsic apoptotic pathway has proven effective in leukemia therapy. For example, artesunate induces mitochondrial apoptosis in drug-resistant T-leukemia cells through a Reactive Oxygen Species (ROS)-dependent mechanism [60]. Therefore, the ability of DF to selectively activate this pathway in leukemic cells highlights its potential as a novel therapeutic strategy for T-ALL.

### 3.4. Drug Repurposing and Therapeutic Implications

Drug repurposing has emerged as an attractive strategy in oncology because it can significantly reduce the time and cost required for clinical translation [61]. In this context, DF represents a promising candidate due to its established safety profile, well-characterized pharmacokinetics, and oral bioavailability [44,62]. Although the plasma concentrations achieved with standard dosing for overactive bladder are substantially lower than the IC_50_ values observed in this study, higher doses may be feasible in the oncology setting given the favorable safety profile of DF [45].

Recent drug repositioning studies have identified several approved compounds with potential activity against acute lymphoblastic leukemia [63], supporting the feasibility of this approach. Furthermore, the non-neuronal cholinergic system has been proposed as a therapeutic target in T-ALL because of the altered cholinergic machinery observed in leukemic cells [23]. To our knowledge, the present study provides the first experimental evidence supporting the antileukemic activity of a selective M3 receptor antagonist in this context.

The selective cytotoxicity exhibited by DF is reminiscent of other agents that exploit cancer-specific vulnerabilities, such as phenothiazines and arabinosylguanine [48,49]. Importantly, identifying glycative stress as a mediator of DF-induced cell death suggests that therapeutic strategies combining inhibition of the cholinergic pathway with disruption of MG detoxification may be a promising approach for T-ALL treatment.

An important limitation regarding the translational potential of DF concerns the relationship between its in vitro potency and clinically achievable exposure. Clinical pharmacokinetic studies have demonstrated that DF exhibits low oral bioavailability (15–19%), extensive plasma protein binding (~98%), and plasma concentrations in the low-nanomolar range at the approved therapeutic doses for overactive bladder, which are substantially lower than the IC_50_ values observed in the present study. Nevertheless, discrepancies between in vitro potency and plasma exposure are common during early drug repurposing studies, particularly in oncology, where tissue accumulation, intracellular drug concentrations, combination therapies, and structural optimization may substantially improve therapeutic efficacy. Therefore, our findings should be interpreted primarily as proof-of-concept supporting mAChR M3/cathepsin signaling as a therapeutic vulnerability in T-ALL and as a rationale for developing more potent DF-based therapeutic strategies [64].

### 3.5. Darifenacin-Mediated Cathepsin Inhibition in Leukemic T Cells

Our findings extend recent observations by Mukhopadhyay et al., who demonstrated that DF inhibits cathepsin B activity in cancer cells [65]. Whereas DF exhibited IC_50_ values of 38.14 µM and 39.96 µM in IMR-32 neuroblastoma and MCF-7 breast cancer cells, respectively [65], T-cell leukemia cells showed greater sensitivity, with IC_50_ values ranging from 10 to 25 µM depending on the cathepsin isoform and cell line. Notably, cathepsin C inhibition exhibited marked selectivity, with Jurkat and MOLT-4 cells showing 4–5-fold greater sensitivity than healthy T lymphocytes, whereas cathepsin B inhibition showed 2.5–3-fold selectivity.

This differential sensitivity was accompanied by elevated basal cathepsin activity in leukemic cells, supporting the notion that T-ALL cells exhibit increased dependence on cathepsin-mediated pathways for survival and proliferation. Indeed, cathepsins B, C, H, L, S, and X are frequently overexpressed in hematological malignancies [38], and cathepsin B overexpression has been associated with poor prognosis in ALL, acute myeloid leukemia (AML), chronic lymphocytic leukemia (CLL), and chronic myeloid leukemia (CML) [38]. These observations are consistent with the concept of oncogenic dependency on cathepsin-mediated signaling pathways [66].

Interestingly, low concentrations of DF induced a transient increase in cathepsin C activity in normal T lymphocytes, suggesting an adaptive hormetic response. Such biphasic responses are common in biological systems [67,68] and may reflect compensatory mechanisms associated with the role of the non-neuronal cholinergic system in immune homeostasis [20,69,70]. The absence of this response in leukemic cells further supports the notion that malignant cells possess a reduced capacity for adaptive compensation [71,72].

Previous studies have shown that DF induces ROS generation and mitochondrial dysfunction in cancer cells [64]. Accordingly, the selective cytotoxicity observed in Jurkat and MOLT-4 cells may result from the combined disruption of cathepsin activity, redox homeostasis, and mitochondrial integrity. This hypothesis is supported by studies demonstrating that the cathepsin B inhibitor z-FA-CMK induces oxidative stress, mitochondrial dysfunction, and caspase-dependent apoptosis in Jurkat cells [73].

The therapeutic implications of these findings are significant. The selective inhibition of cathepsins B and C by DF, together with the established roles of cathepsins in leukemia progression, survival, angiogenesis, and immune evasion [38,74], suggests a therapeutic window that could be exploited clinically. Moreover, the favorable safety profile of DF supports its potential repurposing for leukemia treatment. Because cathepsin inhibition can exacerbate oxidative stress and apoptosis, DF may also prove beneficial when combined with conventional chemotherapy or targeted therapies, potentially overcoming mechanisms of therapeutic resistance [75]. Finally, the complex roles of cathepsins in hematopoiesis and tumor-immune interactions underscore the need to evaluate DF-mediated inhibition of cathepsins in more physiologically relevant models [76,77].

### 3.6. Molecular Docking Analyses Performed with Darifenacin Against Cathepsin B and C

Molecular docking analyses suggest that DF may interact favorably with the catalytic cavities of cathepsins B and C, supporting the hypothesis that DF could act as a non-covalent inhibitor of these proteases. Under the evaluated computational conditions, DF exhibited predicted binding affinities comparable to or greater than those of the co-crystallized inhibitors. However, these observations remain hypothetical and require experimental validation.

The predicted binding modes were characterized by hydrophobic, aromatic, and polar interactions, consistent with the amphipathic nature of DF. In cathepsin B, the predicted interactions with residues of the occluding loop, particularly His110 and His111, are noteworthy, as this region plays a key role in substrate recognition and has been implicated in binding other non-covalent inhibitors [78,79,80]. Although DF lacks the electrophilic groups required for covalent inhibition, its aromatic scaffold may provide sufficient stabilizing interactions within the catalytic cavity [79,81].

Similarly, docking analyses suggest that DF may occupy the catalytic region of cathepsin C through a combination of hydrophobic and polar interactions, potentially interfering with substrate binding or catalysis [52,82,83,84,85]. Recent drug repurposing studies have also identified approved drugs, such as lurasidone and paliperidone, as potential cathepsin inhibitors, supporting the concept that structurally diverse compounds may exhibit unexpected anti-cathepsin activity [86].

These computational analyses provide a plausible structural framework for the inhibitory effects of DF observed in vitro assays and support further biochemical and biophysical studies to validate cathepsins B and C as molecular targets of DF in T-ALL.

### 3.7. Limitations and Future Directions

Several limitations of this study should be acknowledged. First, our findings were obtained using established T-ALL cell lines and healthy primary T lymphocytes from healthy donors; therefore, validation in primary patient samples is necessary to determine whether the differential expression of NNCS components, cathepsin activity, and DF sensitivity are representative of the molecular heterogeneity of T-ALL.

Second, although our results implicate glycative stress, cathepsin inhibition, and activation of the intrinsic apoptotic pathway in DF-induced cytotoxicity, the mechanistic relationships among these processes remain to be elucidated. Future studies should investigate the effects of DF on MG detoxification pathways, including GLO1 activity, redox homeostasis, and calcium-dependent signaling, as well as the contribution of ROS to the induction of apoptosis [58,59]. In addition, although enzymatic assays performed in cellular extracts and molecular docking analyses suggest that cathepsins B and C may represent molecular targets of DF, studies using purified recombinant enzymes, together with biochemical, biophysical, and structural approaches, will be required to validate the direct interaction, define the inhibition mechanism, and confirm the predicted binding modes.

Third, the absence of in vivo studies represents an important limitation. Xenograft and patient-derived models will be necessary to evaluate the antileukemic efficacy, pharmacokinetics, tissue distribution, and safety of DF, as well as its ability to overcome microenvironment-mediated resistance. The potential of combining DF with standard chemotherapy or targeted therapies should also be explored [50].

Despite these limitations, our findings demonstrate that DF exerts selective antileukemic activity associated with glycative stress, cathepsin inhibition, and activation of mitochondrial apoptosis. Given its established safety profile and the growing interest in targeting the non-neuronal cholinergic system in cancer [28], DF warrants further investigation as a potential therapeutic strategy for T-ALL. Structure-activity relationship studies may also facilitate the development of derivatives with improved potency, selectivity, and pharmacokinetic properties [75].

## 4. Materials and Methods

### 4.1. Reagents and General Materials

Phosphate-buffered saline (PBS) and Bradford reagent were obtained from Sigma-Aldrich (St. Louis, MO, USA). Ethylenediaminetetraacetic acid (EDTA)-Vacutainer tubes for blood collection from healthy volunteers were acquired from Becton Dickinson (Franklin Lakes, NJ, USA). Lymphoprep™ Density Gradient Medium for mononuclear cell isolation was purchased from STEMCELL Technologies (Cologne, Germany). Magnetic anti-CD3 microbeads and the MiniMACS™ separation system were from Miltenyi Biotec (Bergisch Gladbach, Germany). RPMI-1640 medium, fetal bovine serum (FBS), penicillin, and streptomycin were acquired from Gibco (Thermo Fisher Scientific, Waltham, MA, USA). Annexin V-FITC and Propidium Iodide (PI) apoptosis detection kits were obtained from BD Biosciences (San Jose, CA, USA). Darifenacin hydrobromide (≥98% purity, high-performance liquid chromatography [HPLC] grade) was purchased from Sigma-Aldrich (St. Louis, MO, USA; catalog number D2689). A 50 mM stock solution was prepared in dimethyl sulfoxide (DMSO; Sigma-Aldrich) and stored at −20 °C as single-use aliquots to avoid repeated freeze–thaw cycles. Working solutions were prepared fresh for each experiment by diluting the stock solution in culture medium to achieve final concentrations ranging from 5 to 100 µM. The final DMSO concentration in all experiments was maintained at ≤0.5% (*v*/*v*), as preliminary experiments showed it did not affect cell viability or apoptosis. Vehicle control treatments contained equivalent concentrations of DMSO. All other reagents were of analytical grade and purchased from Sigma-Aldrich suppliers.

### 4.2. Isolation of Healthy Donor-Derived T Lymphocytes

Primary T lymphocytes from healthy donors were included in comparative assays with leukemic cells. The sample consisted of ten healthy young adults (aged 18 to 22 years) recruited during the second half of 2025, according to the following inclusion and exclusion criteria:

The inclusion criteria were as follows: age between 18 and 22 years, weight between 50 and 80 kg, systolic blood pressure between 90 and 160 mmHg, diastolic blood pressure between 60 and 90 mmHg, pulse rate between 50 and 100 beats per minute, and fasting for 6–8 h. Volunteers were restricted from consuming dietary fats, eggs, milk, or dairy derivatives for 24 h prior to blood donation.

The exclusion criteria were as follows: pregnant or breastfeeding women; individuals who reported influenza, cough, diarrhea or dental infection in the last 14 days; people who had taken medications in the last five days; people who had undergone endodontic treatments, acupuncture or had tattoos or piercings in the last 12 months; those who had undergone surgery in the last six months; those who had been vaccinated in the last 30 days; and those who consumed alcoholic beverages in the 72 h prior to donation.

The study was approved by the Institutional Research, Biosafety, and Ethics Committees (protocol number: 2022/067), in accordance with the Declaration of Helsinki, and written informed consent was obtained from all volunteer donors. Each volunteer donated 20 mL of peripheral blood, from which peripheral blood mononuclear cells (PBMCs) were isolated via density gradient centrifugation using Lymphoprep™ Density Gradient Medium at 2000 rpm for 20 min. The collected mononuclear fraction was resuspended in 1 mL of PBS and incubated with MACS^®^ anti-CD3 MicroBeads (Miltenyi Biotec, Gaithersburg, MD, USA) that target the T lymphocyte cluster of differentiation. Finally, healthy primary T lymphocytes were isolated by positive magnetic selection using the MiniMACS™ Starting Kit (Miltenyi Biotec, Bergisch Gladbach, Germany) following the manufacturer’s protocol.

To ensure optimal physiological status, baseline cell viability and density were strictly verified prior to each experiment using the Trypan blue (Microlab, Monterrey, Mexico) exclusion assay. This quality control protocol was applied to total leukocytes, purified T lymphocytes, Jurkat cells, and MOLT-4 cells, and only viable cells at a density range of 1 × 10^3^ to 1 × 10^6^ cells/mL were used in subsequent assays. The detailed baseline values and concentration-dependent viability profiles for both healthy and leukemic lines are fully documented in Supplementary Figure S2 and Table S1.

### 4.3. Cell Cultures and Regimen Treatments with Darifenacin

The human T-lymphoblast cell lines Jurkat E6-1 (TIB-152) and MOLT-4 (CRL-1582) were obtained from the American Type Culture Collection (ATCC, Rockville, MD, USA) for cell culture assays. Jurkat and MOLT-4 cells were cultured in RPMI-1640 medium (Sigma-Aldrich, St. Louis, MO, USA) supplemented with 10% (*v*/*v*) fetal bovine serum, 2 mM L-glutamine, 1 mM sodium pyruvate, 100 U/mL penicillin, and 100 μg/mL streptomycin. Healthy primary T lymphocytes were cultured under similar conditions, as RPMI is a suitable culture medium for T cells. To ensure that cytotoxicity assays were conducted under metabolic and proliferative conditions comparable to those of the leukemic cell lines, healthy T lymphocytes isolated from peripheral blood were not evaluated in a resting state. Prior to DF treatment, these cells were activated in vitro by adding phytohemagglutinin (PHA) at 5 µg/mL for 72 h to induce proliferation and entry into the cell cycle.

For all experiments, Jurkat and MOLT-4 cells from passages 2 to 5, as well as freshly isolated healthy T lymphocytes, were used. The cells were cultured at 37 °C in a humidified atmosphere with 5% CO_2_. Following culture, the cells were centrifuged at 2500 rpm for 5 min and washed with PBS.

Jurkat and MOLT-4 cells, as well as healthy T lymphocytes, were exposed to increasing concentrations of DF (0–1000 μM) to assess cell viability and other biochemical parameters. In all control conditions (absence of DF), cells were incubated with the highest DMSO concentration used as the vehicle control (0.5% *v*/*v*).

Trypan Blue Exclusion Assay: Cells were seeded at a density of 1 × 10^5^ cells per well in a final volume of 1 mL in 24-well plates and exposed to DF at 0 to 50 μM for 24 h under the specified culture conditions. After the incubation period, the cells were collected, centrifuged, resuspended in PBS, and washed three times. Cell density and membrane-integrity viability were determined using a hemocytometer via the Trypan blue exclusion assay. For each individual treatment, cell viability was expressed as the percentage of total counted cells minus the percentage of dead cells.

MTT Metabolic Viability Assay: Metabolic cell viability was assessed using the 3-(4,5-dimethylthiazol-2-yl)-2,5-diphenyltetrazolium bromide (MTT) assay. Briefly, following the 24 h treatment with DF (0–50 μM), Jurkat cells, MOLT-4 cells, or healthy T lymphocytes (1 × 10^5^ cells) were incubated in 100 μL of PBS per well in a 96-well plate. After the addition of MTT, cells were incubated for 4 h in the dark. The resulting formazan crystals were dissolved in DMSO, and absorbance was monitored at 570 nm using an Epoch microplate spectrophotometer (BioTek, Winooski, VT, USA). Results represent the average of three independent experiments, presented as percentage viability relative to the pharmacological treatment, with the viability of untreated cells set at 100%.

### 4.4. Western Blot Assays for mAChR M3, ChAT, AChE, Pro- and Anti-Apoptotic Proteins

Jurkat cells, MOLT-4 cells, and healthy T lymphocytes were cultured at a density of 5 × 10^6^ cells/well in 6-well plates and treated with increasing concentrations of DF (0, 30, and 50 μM) for 24 h. After treatment, cells were harvested by centrifugation and lysed in ice-cold RIPA buffer supplemented with protease inhibitors. Lysates were stored at −70 °C until further analysis. Protein concentration was determined using the Bradford assay, and 100 µg of total protein per sample was loaded on 12% SDS-PAGE gels. Electrophoresis was carried out at 100 V, followed by 150 V for 2 h at 4 °C. Proteins were transferred to PVDF membranes, which were then blocked in 5% non-fat milk in TBS-Tween-20 for 1 h at room temperature. Membranes were incubated overnight at 4 °C with primary antibodies against mAChR M3 (H-4), ChAT (E-7), AChE (A-11), Bax (4H32), Bcl-2 (C-2), Caspase-3 (E-8) (Santa Cruz Biotechnology, Dallas, TX, USA), or anti-β-Actin (ab8227; Abcam, Cambridge, UK), diluted 1:1000 in TBS-Tween-20 with 1% BSA. After washing, membranes were incubated for 1 h with HRP-conjugated secondary antibodies (1:3000), and bands were detected using the Clarity Western ECL substrate (Bio-Rad, Hercules, CA, USA).

Prior to incubation with the anti-β-Actin antibody (ab8227), membranes previously probed for mAChR M3 (H-4), Choactase (E-7), AChE (A-11), Bax (4H32), Bcl-2 (C-2), and Caspase-3 (E-8) were stripped using Restore™ Western Blot Stripping Buffer (Thermo Fisher Scientific, Waltham, MA, USA) for 15 min at room temperature with gentle agitation, followed by three washes with TBS-Tween-20 and re-blocking in 5% non-fat milk in TBS-Tween-20 for 1 h at room temperature before proceeding with β-Actin detection. Band intensity was analyzed using the Molecular Imager^®^ Gel Doc™ XR+ system (Bio-Rad), and densitometry was performed with Image Studio 4.0 software (LI-COR Biotechnology, Lincoln, NE, USA) after normalization to β-Actin.

### 4.5. Apoptosis Detection by Flow Cytometry

To evaluate the effect of DF, Jurkat and MOLT-4 cells, as well as healthy T lymphocytes, were seeded in 6-well culture plates at a density of 1 × 10^6^ cells per well in 2 mL of RPMI-1640 supplemented medium. Cells were treated for 24 h with DF at concentrations of 0, 30, and 50 μM. A vehicle control group receiving 0.5% (*v*/*v*) DMSO was included to assess the baseline conditions.

Following treatment, total cell counts were quantified using a Neubauer hemocytometer (Marienfeld, Lauda-Königshofen, Germany). For apoptotic analysis, cells were collected, washed twice with cold PBS, and resuspended in binding buffer to a final concentration of 1 × 10^6^ cells/mL. Annexin V-FITC and PI staining were performed using a commercial apoptosis detection kit (BD Biosciences), according to the manufacturer’s guidelines.

To establish compensation controls for flow cytometric gating and to distinguish between apoptotic and necrotic populations, additional cell samples were included: (1) untreated and unstained cells, (2) cells treated with 50 µM hydrogen peroxide for 5 h to induce early apoptosis, and (3) cells treated with 500 µM hydrogen peroxide for 5 h to induce necrosis. These controls were used to define fluorescence spillover parameters and generate the calculation matrix.

Stained cells were analyzed using a Guava^®^ easyCyte™ flow cytometer (Cytek^®^ Biosciences, Fremont, CA, USA). For each sample, exactly 30,000 events were acquired at a medium flow rate (<800 events/s). To minimize the inclusion of background noise and small debris during data acquisition, an electronic forward scatter (FSC) threshold was strictly set to 3000. All data were acquired and processed with InCyte™ Software v3.1 (Merck Millipore, Billerica, MA, USA).

The standard gating strategy consisted of drawing a primary morphological gate on the main cell population in a forward scatter versus side scatter (SSC) cytogram. This step effectively excluded cell debris (characterized by low FSC and SSC signals) as well as artifactual events with disproportionately large size and granularity. On average, this primary morphological gate encompassed approximately 70% of the total acquired events, yielding a consistent baseline of ~21,000 analyzed events per sample for downstream fluorescence evaluation.

Due to the specific hardware configuration and standard acquisition protocols of the Guava easyCyte system, pulse geometry parameters (FSC-Area, FSC-Height, and FSC-Width) were not recorded; consequently, formal doublet discrimination (singlet selection) could not be integrated into the gating hierarchy. Nevertheless, given the homogeneous single-cell suspension nature of these lymphoblastoid lines and the direct evaluation of a single target population via Annexin V/PI fluorescence, this instrument-specific limitation exerts a negligible impact on the relative interpretation and validity of the apoptotic indices.

Percentages of viable (Annexin V^−^/PI^−^), early apoptotic (Annexin V^+^/PI^−^), late apoptotic (Annexin V^+^/PI^+^), and necrotic (Annexin V^−^/PI^+^) cells were determined within this gated population. All treatments were conducted in triplicate, and data are presented as mean values ± SD from three independent biological experiments (*n* = 3).

### 4.6. Quantification of MG and AGEs in Darifenacin-Treated Cells

To assess intracellular levels of MG and AGEs, Jurkat and MOLT-4 cells, as well as healthy T lymphocytes, were exposed to DF at concentrations of 0, 30, and 50 μM for 24 h. After treatment, 1 × 10^6^ cells per condition were harvested by centrifugation at 2500 rpm for 10 min at 4 °C. The resulting pellets were washed three times with cold PBS to remove residual media and any metabolites. Cells were then resuspended in PBS at a concentration of 5 × 10^6^ cells/200 µL and subjected to five cycles of rapid freezing in liquid nitrogen (10 s), followed by thawing at 37 °C (1–2 min) to ensure complete cell lysis.

To precipitate proteins and prepare samples for MG quantification, perchloric acid was added to each lysate to a final concentration of 10%. Samples were kept on ice for 10 min, then centrifuged at 12,000 rpm for 10 min at 4 °C. The supernatant containing free intracellular MG was collected and stored at −70 °C until analysis.

Intracellular MG levels were determined via a colorimetric assay based on the reaction of MG with 2,4-dinitrophenylhydrazine (DNPH). The standard calibration curve consisted of tubes containing 0–10 µM MG, which were incubated with 0.2 mM DNPH in a HCl-ethanol mixture (12:88 *v*/*v*) at 42 °C for 45 min. After cooling the samples to room temperature for 5 min, absorbance was measured at 432 nm using a microplate reader (Epoch, BioTek, Winooski, VT, USA). The MG content in cell samples was calculated from the standard curve using an extinction coefficient ε = 33,600 M^−1^ cm^−1^ for the MG-bis-DNPH adduct. Results were expressed as µM MG per 1 × 10^6^ cells and represent the average of three independent experiments.

For AGEs quantification, aliquots of the same cell lysates were adjusted to a protein concentration of 1 mg/mL and quantified using the Bradford assay. Samples were then diluted 1:100 and analyzed using a commercial ELISA kit for AGEs (MyBioSource, San Diego, CA, USA) according to the manufacturer’s instructions. After incubation with avidin-HRP conjugates and addition of the 3,3′,5,5′-tetramethylbenzidine (TMB) substrate, absorbance was measured at 450 nm within 10 min. AGEs concentrations were derived from a standard curve ranging from 0 to 200 ng/mL and expressed as µg AGEs per mL of cell lysate. Each condition was tested in triplicate, and the values presented reflect the mean ± standard deviation from at least three independent experiments.

### 4.7. Hydrolysis Assays with Artificial Fluorogenic Substrates of Cathepsins C and B in Healthy T Lymphocytes and Leukemic Cells

The hydrolytic activity of cathepsin B from each cell lysate was determined using Z-Arg-Arg-7-amido-4-methylcoumarin hydrochloride substrate (Sigma-Aldrich, Cat. No. C5429-5MG). The emitted fluorescence was measured at 25 °C using an LS55 spectrofluorometer (PerkinElmer, Shelton, CT, USA) in a quartz cuvette; the excitation wavelength was 360 nm, and the emission wavelength was 460 nm. In both cases, the buffer’s background fluorescence was subtracted from the sample reading. The reaction mixtures contained 100 mM MES (pH 6.0), 60 μM Z-R-R-7-AMC, and 100 μg/mL total protein from DF-treated cell lysates, in a final volume of 600 μL [87].

The Cathepsin C activity in each sample obtained after DF treatment was monitored and determined by spectrophotometry at 412 nm in a final volume of 600 μL. The activity was determined and incorporated into polycarbonate cells with a path length of 1 cm. The samples, in 100 mM MES buffer at pH 6, contained 500 μM of the synthetic chromogenic substrate Gly-Phe-p-nitroanilide (Sigma, Cat. No. G0142) and 100 μg of cell lysate protein. The increase in absorbance at 412 nm was monitored at 25 °C. Following the addition of the protein to the sample, proteolysis of the synthetic substrate GP-p-NA released the p-nitroanilide group, generating a change in absorbance (ΔAbs). The blank readings corresponded to the baseline absorbance reading recorded before protein addition. The absorptivity coefficient (ε) of p-nitroanilide was used to obtain the activity of each fraction [88].

### 4.8. Molecular Docking

To investigate the potential molecular interactions between DF and Cathepsins, in silico molecular docking analyses were performed using the crystallographic structures of human cathepsin B (PDB ID: 8B4T) [51] and human cathepsin C (PDB ID: 6IC7) [52], retrieved from the Protein Data Bank (PDB; Research Collaboratory for Structural Bioinformatics, New Brunswick, NJ, USA) [53].

Prior to docking simulations, protein structures were prepared by ligand removal, protonation, and energy minimization before docking simulations. Docking calculations were performed using the CB-Dock server (Sichuan University, Chengdu, China) under standard conditions [54]. The ligand structure of DF was retrieved from the PubChem database (National Center for Biotechnology Information, Bethesda, MD, USA; (https://pubchem.ncbi.nlm.nih.gov/) (accessed on 30 September 2025)).

Docking calculations were performed using the CB-Dock server with default parameters. The docking protocol evaluated ligand positioning within the catalytic cavities of both cathepsins and generated predicted binding affinities expressed in kcal/mol. The resulting docking poses were ranked by binding energy, and the conformations with the lowest predicted free energy values were selected for subsequent interaction analyses.

Protein-ligand interactions were analyzed by examining distances and spatial orientations between DF and residues lining the catalytic clefts. Molecular representations were generated using PyMOL Molecular Graphics System version 2.5.0 (Schrödinger, LLC, New York, NY, USA).

### 4.9. Statistical Analysis

The data are presented as mean ± standard deviation (SD). Statistical analyses were performed using NCSS v22.09 (NCSS, LLC, Kaysville, UT, USA). Multiple group comparisons were performed using one-way analysis of variance (ANOVA) followed by Tukey’s post hoc test for normally distributed data. A *p*-value < 0.05 was considered statistically significant.

## 5. Conclusions

Our results demonstrate the in vitro efficacy of DF against T-ALL cell lines. Although DF requires high micromolar concentrations (30–50 µM) to exert its antileukemic effect in vitro, the lack of significant cytotoxicity against healthy activated T lymphocytes indicates substantial cytotoxic selectivity. This favorable therapeutic window suggests a promising safety margin and provides a solid baseline for the development of future in vivo dose-escalation studies.

Furthermore, this study provides initial in vitro evidence suggesting that DF exhibits this selective cytotoxicity through multiple interconnected mechanisms, including M3 receptor blockade, induction of glycative stress, and activation of intrinsic apoptosis. Crucially, this multi-targeted profile was experimentally confirmed by enzymatic assays and supported by computational molecular docking, demonstrating direct, simultaneous inhibition of cathepsins B and C and thereby validating a dual mechanism of action.

Altogether, these findings warrant further investigation of DF as a promising candidate for repurposing in T-ALL and highlight the potential of simultaneously targeting the NNCS and lysosomal cathepsins in hematological malignancies.

## Data Availability

The original contributions presented in this study are included in the article/Supplementary Material. Further inquiries can be directed to the corresponding authors.

## Supplementary Materials

The following supporting information can be downloaded at: https://www.mdpi.com/article/10.3390/ijms27146417/s1.
