# Supplementary material for: Evaluation of Darifenacin for T-Cell Acute Lymphoblastic Leukemia: Selective Targeting of the Non-Neuronal Cholinergic System and Lysosomal Cathepsins
Source: Int J Mol Sci. 2026 Jul 19;27(14):6417. doi: 10.3390/ijms27146417 (PMC13410119; doi:10.3390/ijms27146417)
Supplement: Supplementary file 1 [file ijms-27-06417-s001.zip › ijms-4399559-supplementary.pdf]

International Journal of *Molecular Sciences*

MDPI

Article

Evaluation of Darifenacin for T-Cell Acute Lymphoblastic Leukemia: Selective Targeting of the Non-Neuronal Cholinergic System and Lysosomal Cathepsins

Luis A. Flores-López [1,*], Yoalli Martínez-Pérez [2], Ignacio De la Mora-De la Mora [3], Gabriela López-Herrera [4], Saúl Gómez-Manzo [5], Itzhel García-Torres [3], Beatriz Hernández-Ochoa [6] and Sergio Enríquez-Flores [3,*]

1 SECIHTI-Laboratorio de Biomoléculas y Salud Infantil, Instituto Nacional de Pediatría, Mexico City 04530, Mexico
2 Escuela de Medicina y Ciencias de la Salud, Tecnológico de Monterrey, Mexico City 14380, Mexico; yoalli.martinez@tec.mx
3 Laboratorio de Biomoléculas y Salud Infantil, Instituto Nacional de Pediatría, Mexico City 04530, Mexico; ignaciodelamora@ciencias.unam.mx (I.D.l.M.-D.l.M.); garcia.itzhel@gmail.com (I.G.-T.)
4 Laboratorio de Inmunodeficiencias, Instituto Nacional de Pediatría, Mexico City 04530, Mexico; lohegabyqbp@gmail.com
5 Laboratorio de Bioquímica Genética, Instituto Nacional de Pediatría, Mexico City 04530, Mexico; saulmanzo@ciencias.unam.mx
6 Laboratorio de Investigación en Ciencias Ómicas y Epidemiología Microbiana, Hospital Infantil de México Federico Gómez, Secretaría de Salud, Mexico City 06720, Mexico; beatrizhb_16@comunidad.unam.mx
* Correspondence: lfloresl@secihti.mx (L.A.F.-L.); sergioenriquez@ciencias.unam.mx (S.E.-F.); Tel.: +52-5510840900 (ext. 1425 or 1726)

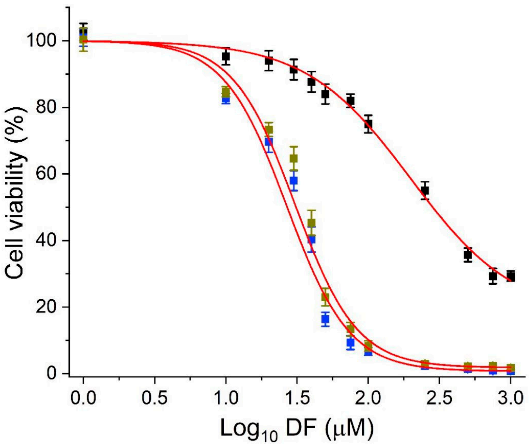

Cell type	IC_{50} (μM)	95% CI (μM)
T-lymphocytes	197.9	148.9–262.9
Jurkat	26.7	23.2–30.7
MOLT-4	30.5	27.0–34.4

Supplementary Figure S1. Semi-logarithmic dose-response curves for cell viability following DF treatment. T lymphocytes (black), Jurkat cells (blue), and M⊚ LT-4 cells (green) (1 × 10^6 cells/mL) were exposed for 24 h to increasing concentrations of DF (0, 10, 20, 30, 40, 50, 75, 100, 250, 500, 750, and 1000 μM). Cell viability was determined by the MTT assay and is expressed as the percentage of viable cells relative to the untreated control (100%). Data represent the mean ± Standard Deviation (SD) of three independent biological replicates. Dose-response curves were generated by nonlinear regression using a four-parameter logistic (4PL) model and used to determine IC_{50} values. The accompanying table summarizes the estimated IC_{50} values and their corresponding 95% confidence intervals (95% CI). The corresponding nonlinear regression analyses yielded R^2 values of 0.9955 for normal T lymphocytes, 0.9899 for Jurkat cells, and 0.9919 for M⊚ LT-4 cells.

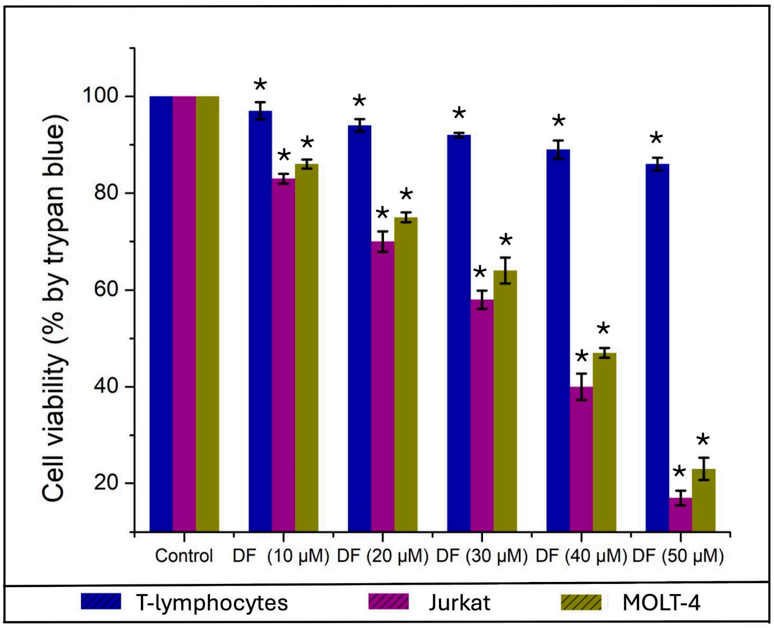

Supplementary Figure S2. Concentration-dependent cytotoxicity and screening selectivity of Darifenacin via Trypan Blue exclusion. Grouped bar chart illustrating the comparative cell viability of healthy activated T lymphocytes, Jurkat cells, and M⊚ LT-4 cells after 24 h of treatment with Darifenacin (0–50 μM). The marked divergence between the healthy T cell profile and the leukemic cell lines underscores substantial cytotoxic selectivity and a favorable therapeutic window at mid- to high-micromolar concentrations. Data represent three independent biological replicates (mean ± SD). Statistical analysis was performed using one-way Analysis of Variance (AN⊚ VA) followed by Tukey's post hoc test, with statistical significance defined as $p < 0.05$ (*). Exact numerical values and statistical parameters are compiled in Supplementary Table S1.

Supplementary Table S1. Quantitative cell viability profile of T lymphocytes and leukemic T cells treated with DF. Cell viability was evaluated using the Trypan Blue exclusion assay after 24 h of exposure to different concentrations of DF (0, 10, 20, 30, 40, and 50 μM). Data are expressed as the mean percentage of viable cells ± SD from three independent biological replicates. The high baseline viability (>98%) in untreated controls confirms the optimal physiological status of the cultures prior to experimental procedures.

**Concentration (μM)**	**T lymphocytes (%)**	**Jurkat cells (%)**	**MOLT-4 cells (%)**
0 (Control)	100.00 ± 0.00	98.63 ± 0.16	98.91 ± 0.41
10	96.23 ± 2.18	80.72 ± 0.59	84.35 ± 1.43
20	91.51 ± 1.05	68.58 ± 1.44	73.39 ± 0.41
30	90.09 ± 0.63	55.27 ± 0.72	62.06 ± 2.19
40	86.27 ± 0.69	37.68 ± 1.17	45.40 ± 0.92
50	84.00 ± 1.90	14.79 ± 0.93	20.88 ± 0.60

Supplementary Table S2. Densitometric values of NNCS components following DF treatment. Protein expression levels of mAChR M3, ChAT, and AChE normalized to β-actin. Values are presented as mean ± SD of three independent experiments.

**Target Protein**	**Cell Line**	**Control (0 μM)**	**DF 30 μM**	**DF 50 μM**
**mAChR M3**	T lymphocytes	0.552 ± 0.006	0.524 ± 0.023	0.504 ± 0.038
	Jurkat	0.869 ± 0.015	0.565 ± 0.022	0.337 ± 0.019
	M◎ LT-4	0.855 ± 0.013	0.600 ± 0.020	0.387 ± 0.010
**ChAT**	T lymphocytes	0.573 ± 0.009	0.560 ± 0.015	0.531 ± 0.024
	Jurkat	0.937 ± 0.023	0.414 ± 0.018	0.238 ± 0.031
	M◎ LT-4	0.914 ± 0.026	0.471 ± 0.022	0.299 ± 0.016
**AChE**	T lymphocytes	0.627 ± 0.019	0.629 ± 0.018	0.640 ± 0.015
	Jurkat	0.362 ± 0.030	0.506 ± 0.012	0.651 ± 0.013
	M◎ LT-4	0.395 ± 0.017	0.523 ± 0.015	0.668 ± 0.023

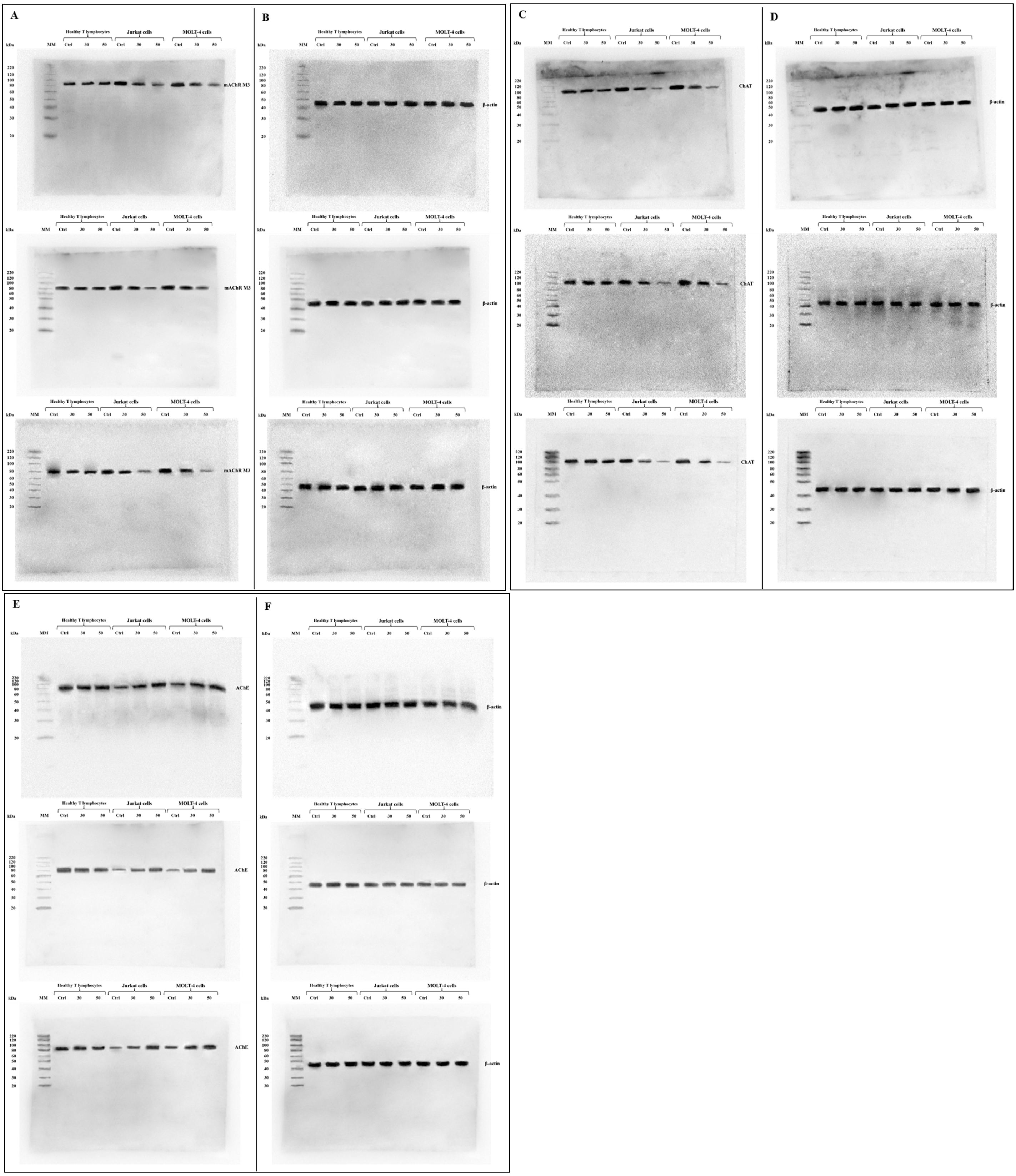

Supplementary Figure S3. Uncropped full-length Western blots correspond to Figure 2. Panels A and B show the uncropped blots for all three independent biological replicates (n = 3) of mAChR M3 and its respective β-actin loading control. Panels C and D show the uncropped blots for the three independent biological replicates of ChAT and β-actin. Panels E and F show the uncropped blots for the three independent biological replicates of AChE and β-actin. Molecular weight markers (kDa) are indicated on the side of each blot, and target bands are highlighted.

Supplementary Table S3. Flow cytometry of healthy T-Lymphocytes, Jurkat cells, and M◎LT-4 cells after DF treatment. Values represent the mean ± SD of three independent biological experiments (*n* = 3).

**Healthy T-lymphocytes**				
**Group**	**Viability (%)**	**Early apoptosis (%)**	**Late apoptosis (%)**	**Necrosis (%)**
	**Mean ± SD**	**Mean ± SD**	**Mean ± SD**	**Mean ± SD**
**Control**	99.77 ± 0.04	0.09 ± 0.05	0.06 ± 0.02	0.08 ± 0.01
**30 μM**	95.67 ± 0.24	1.34 ± 0.09	1.71 ± 0.05	1.28 ± 0.10
**50 μM**	88.42 ± 1.43	3.80 ± 0.40	4.46 ± 0.59	3.32 ± 0.46
**Jurkat cells**				
**Group**	**Viability (%)**	**Early apoptosis (%)**	**Late apoptosis (%)**	**Necrosis (%)**
	**Mean ± SD**	**Mean ± SD**	**Mean ± SD**	**Mean ± SD**
**Control**	99.79 ± 0.02	0.09 ± 0.01	0.05 ± 0.03	0.07 ± 0.02
**30 μM**	57.78 ± 1.38	16.78 ± 0.42	19.70 ± 0.65	5.74 ± 0.33
**50 μM**	14.84 ± 0.34	27.13 ± 0.22	46.32 ± 0.47	11.71 ± 0.49
**MOLT-4 cells**				
**Group**	**Viability (%)**	**Early apoptosis (%)**	**Late apoptosis (%)**	**Necrosis (%)**
	**Mean ± SD**	**Mean ± SD**	**Mean ± SD**	**Mean ± SD**
**Control**	99.78 ± 0.08	0.08 ± 0.04	0.06 ± 0.04	0.08 ± 0.01
**30 μM**	61.84 ± 0.69	15.41 ± 0.51	17.63 ± 0.54	5.13 ± 0.11
**50 μM**	20.80 ± 0.55	23.30 ± 0.55	41.28 ± 0.78	14.62 ± 0.63

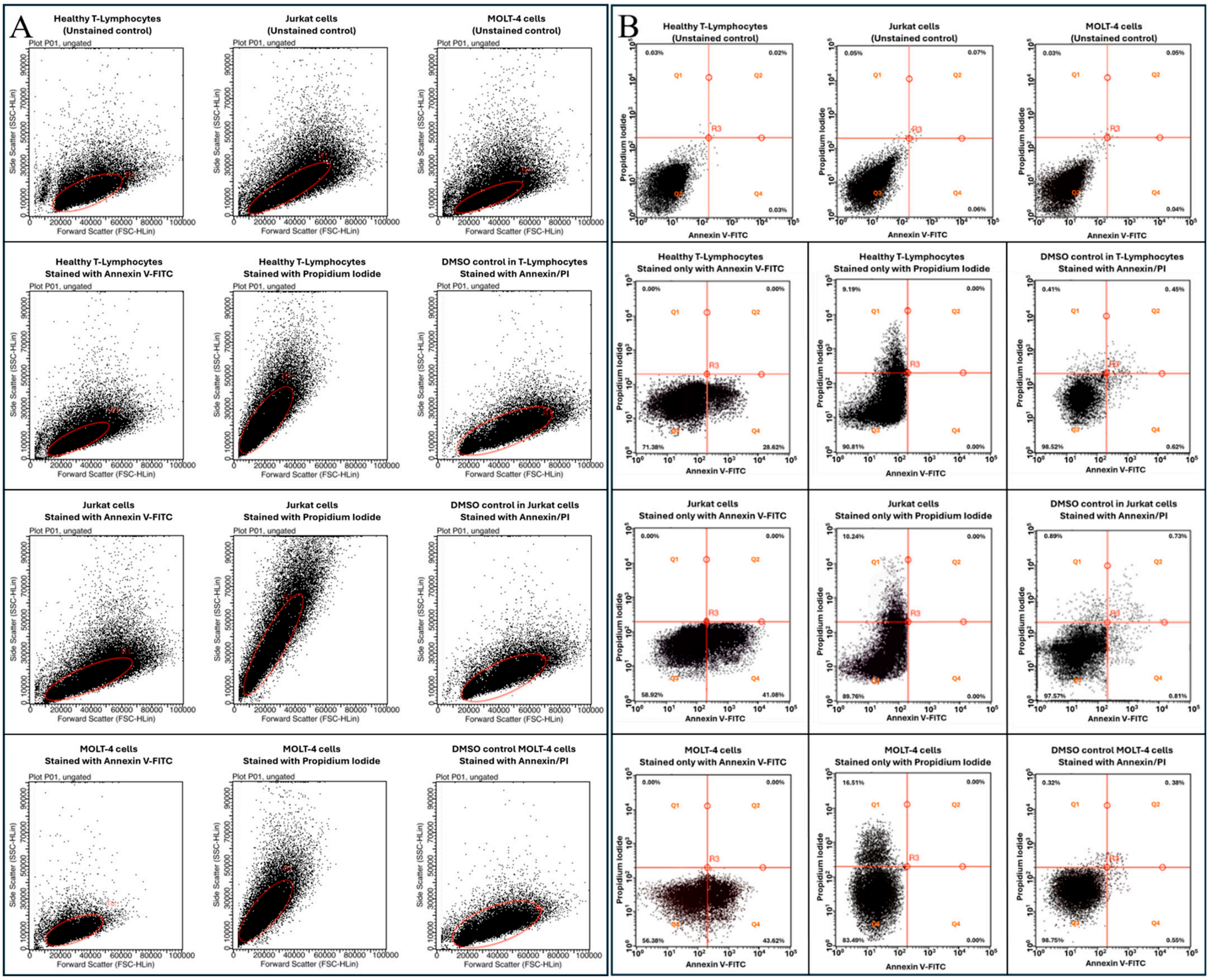

Supplementary Figure S4. Sequential gating strategy, compensation matrix, and baseline validation for apoptosis analysis. (A) Representative forward scatter (FSC-HLin) versus side scatter (SSC-HLin) dot plots displaying the primary gating strategy for healthy primary T-lymphocytes, Jurkat cells, and M⊚ LT-4 cells across controls. The red elliptical gate (R1) was systematically applied to isolate the intact cell population and exclude cellular debris or aggregates based on 30,000 acquired events per sample. (B) Fluorescence intensity quadrant plots of the gated R1 population. Row 1 shows unstained controls used to set baseline PMT voltages and autofluorescence grids. Columns 1 and 2 (Rows 2–4) represent single-stained controls for Annexin V-FITC and PI, used to determine spectral compensation parameters. Column 3 demonstrates double-stained vehicle controls (DMS⊚ ≤ 0.5%), confirming that the solvent does not induce baseline cytotoxicity or apoptotic alterations in any cell model. Quadrants are defined as Q1: necrosis; Q2: late apoptosis; Q3: viable; and Q4: early apoptosis.

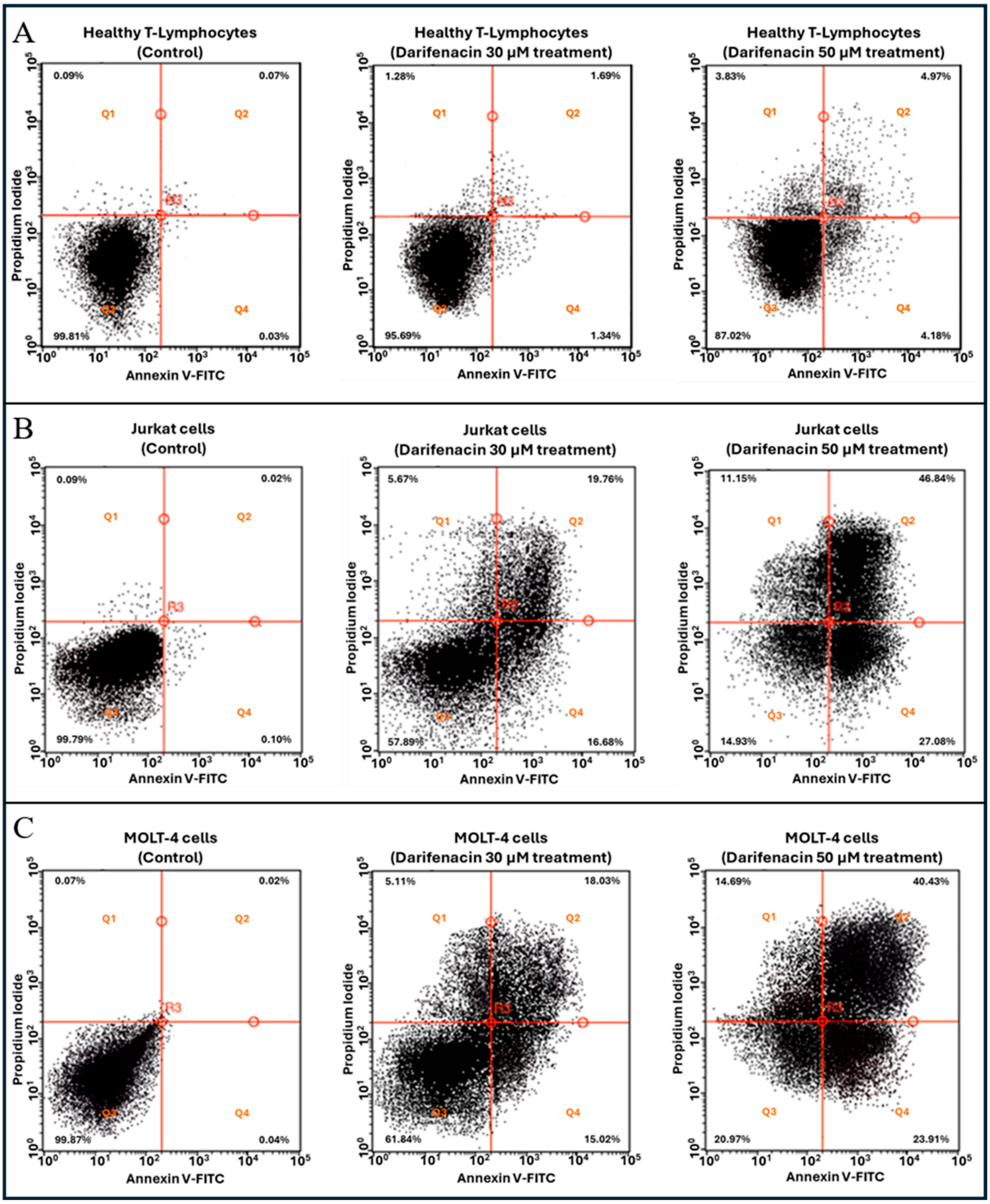
A
Healthy T-Lymphocytes (Control)
0.09%
0.07%
99.81%
0.03%
Healthy T-Lymphocytes (Darifenacin 30 μM treatment)
1.28%
1.69%
95.69%
1.34%
Healthy T-Lymphocytes (Darifenacin 50 μM treatment)
3.83%
4.97%
87.02%
4.18%
B
Jurkat cells (Control)
0.09%
0.02%
99.79%
0.10%
Jurkat cells (Darifenacin 30 μM treatment)
5.67%
19.76%
57.89%
16.68%
Jurkat cells (Darifenacin 50 μM treatment)
11.15%
46.84%
14.93%
27.08%
C
MOLT-4 cells (Control)
0.07%
0.02%
99.87%
0.04%
MOLT-4 cells (Darifenacin 30 μM treatment)
5.11%
18.03%
61.84%
15.02%
MOLT-4 cells (Darifenacin 50 μM treatment)
14.69%
40.43%
20.97%
23.91%
Q1
Q2
Q3
Q4
R3
Propidium Iodide
Annexin V-FITC

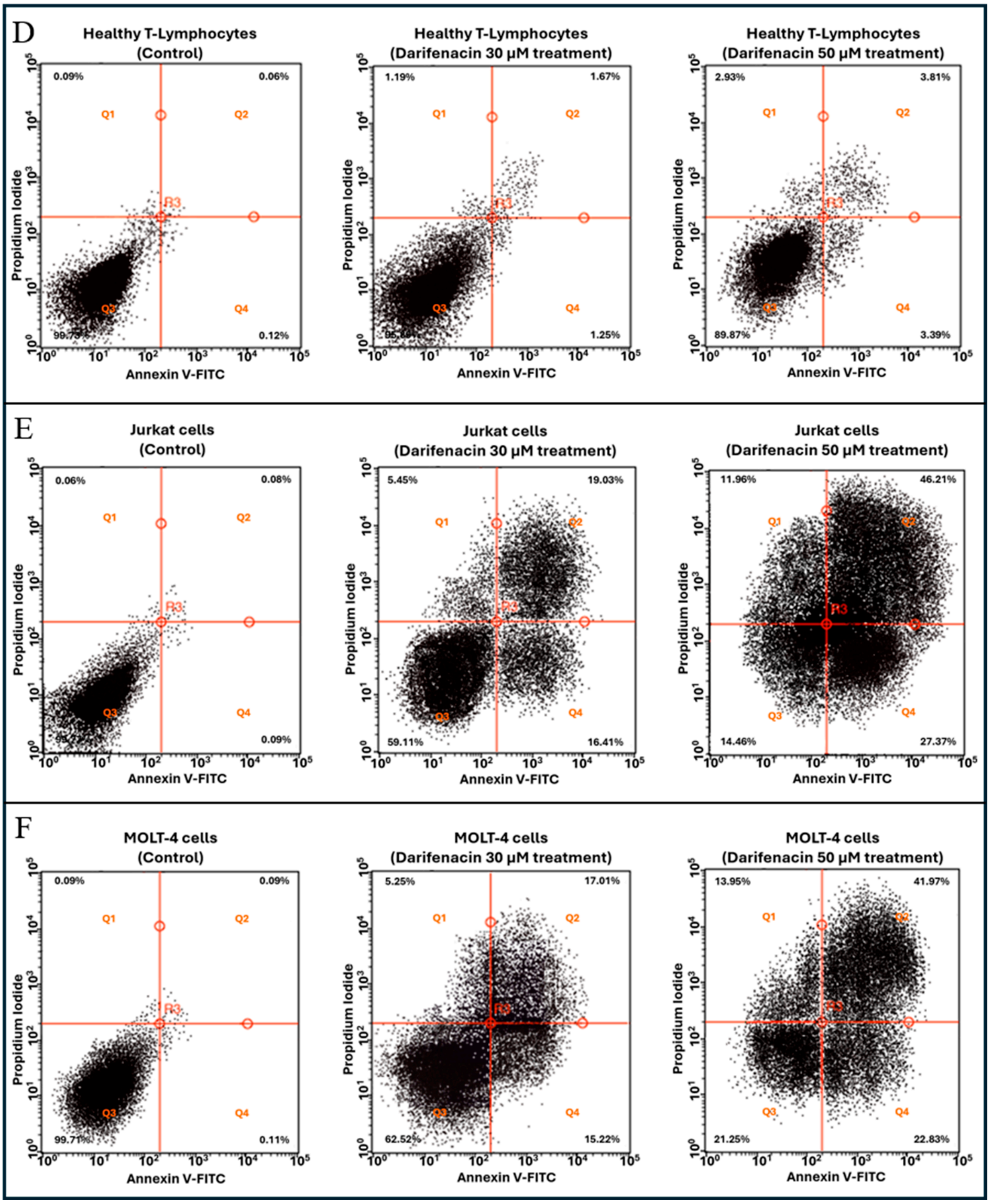

Supplementary Figure S5. Additional representative Annexin V/PI flow cytometry plots following DF treatment. Flow cytometry dot plots of Annexin V–FITC/PI staining in (A and D) Healthy T lymphocytes, (B and E) Jurkat cells, and (C and F) M◎LT-4 cells treated with increasing concentrations of DF (control [0], 30, and 50 µM) for 24 h at 37 °C. Cells were classified as viable (Annexin V$^-$/PI$^-$), early apoptotic (Annexin V$^+$/PI$^-$), late apoptotic (Annexin V$^+$/PI$^+$), or necrotic (Annexin V$^-$/PI$^+$). The data shown correspond to two additional experiments from Figure 3 and Supplementary Table S3.

Supplementary Table S4. Densitometric values of apoptotic markers following Darifenacin treatment. Protein expression levels normalized to β-actin. Bax/Bcl-2 ratios were calculated directly from the respective protein means. Values are presented as mean ± SD of three independent experiments.

Target Protein	Cell Line	Control (0 μM)	DF 30 μM	DF 50 μM
**Pro-caspase-3**	Healthy T lymphocytes	0.570 ± 0.013	0.531 ± 0.016	0.488 ± 0.016
	Jurkat	0.543 ± 0.020	0.313 ± 0.027	0.117 ± 0.017
	M◎ LT-4	0.553 ± 0.020	0.322 ± 0.017	0.143 ± 0.013
**Active Caspase-3**	Healthy T lymphocytes	0.006 ± 0.001	0.047 ± 0.001	0.092 ± 0.004
	Jurkat	0.060 ± 0.010	0.309 ± 0.010	0.507 ± 0.023
	M◎ LT-4	0.066 ± 0.022	0.301 ± 0.025	0.479 ± 0.023
**Bax**	Healthy T lymphocytes	0.237 ± 0.003	0.315 ± 0.012	0.391 ± 0.012
	Jurkat	0.260 ± 0.016	0.585 ± 0.036	0.832 ± 0.031
	M◎ LT-4	0.253 ± 0.024	0.557 ± 0.025	0.802 ± 0.032
**Bcl-2**	Healthy T lymphocytes	0.848 ± 0.011	0.767 ± 0.021	0.690 ± 0.012
	Jurkat	0.833 ± 0.034	0.533 ± 0.029	0.223 ± 0.023
	M◎ LT-4	0.849 ± 0.024	0.550 ± 0.029	0.253 ± 0.022
**Bax/Bcl-2 Ratio**	Healthy T lymphocytes	0.279	0.411	0.568
	Jurkat	0.312	1.098	3.736
	M◎ LT-4	0.298	1.012	3.168

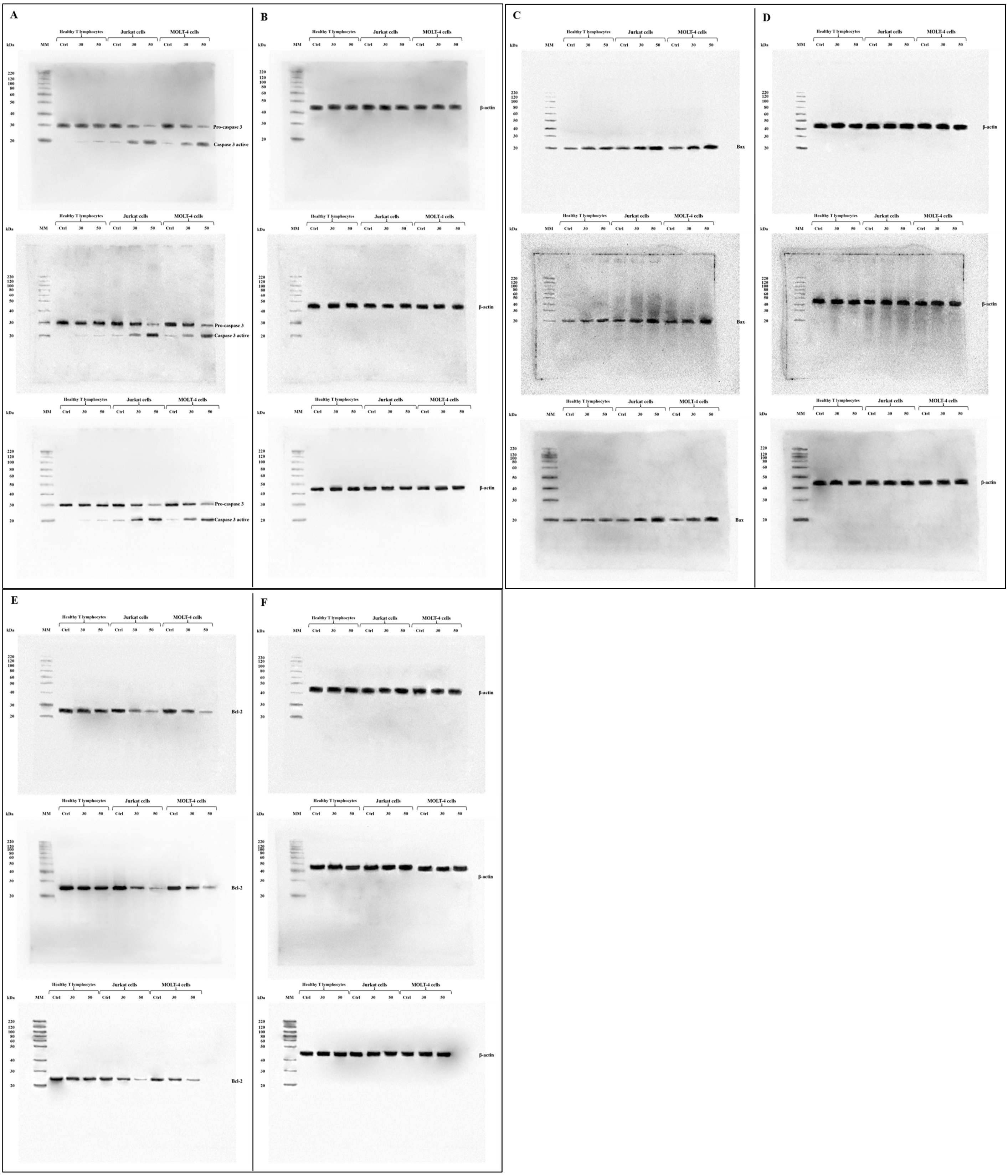

Supplementary Figure S6. Uncropped full-length Western blots corresponding to Figure 5. Panels A and B show the uncropped blots for all three independent biological replicates (n = 3) of pro-caspase-3/caspase-3 activity and their respective β-actin loading controls. Panels C and D show the uncropped blots for the three independent biological replicates of Bax and β-actin. Panels E and F show the uncropped blots for the three independent biological replicates of Bcl-2 and β-actin. Molecular weight markers (kDa) are indicated on the side of each blot, and target bands are highlighted.

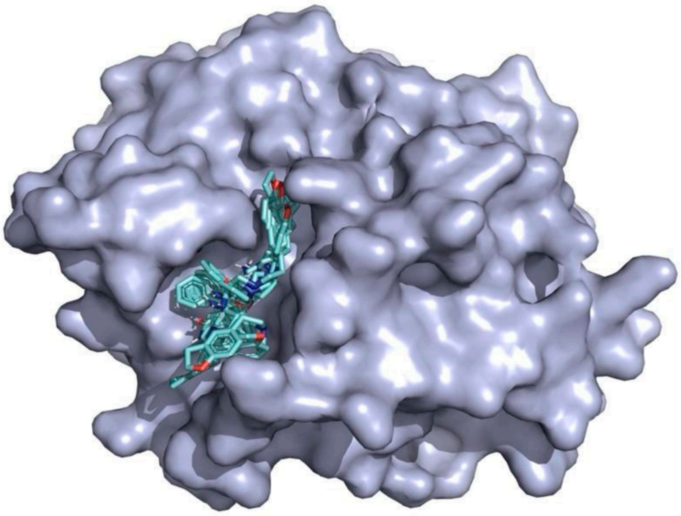

Vina score (kcal/mol) (Darifenacin)	Cavity
-8.6	Active site
-7	Other
-7	Other
-6.5	Other
-6.5	Other

Vina score (kcal/mol) (Inhibitor 7)	Cavity size
-6.3	Active site
-5.7	Other
-5.6	Other
-5.1	Other
-5.1	Other

Supplementary Figure S7. Molecular docking analysis of DF within the catalytic cavity of Cathepsin B. (Left) Surface representation of Cathepsin B showing the different docking conformations predicted for DF within the active-site cleft. The ligand is displayed in multiple orientations to illustrate the preferential occupation of the catalytic pocket and the consistency of ligand accommodation across docking simulations. (Right) Comparative tables showing the predicted binding affinity values obtained for Cathepsin B complexed with DF and with the co-crystallized reference inhibitor (Inhibitor 7). DF exhibited a more favorable predicted interaction energy (-8.6 kcal/mol) than the reference inhibitor (-6.3 kcal/mol), suggesting greater energetic stabilization within the enzyme's catalytic region. Molecular representations were generated using PyM⊚ L, and docking calculations were performed using the CD-Dock server.

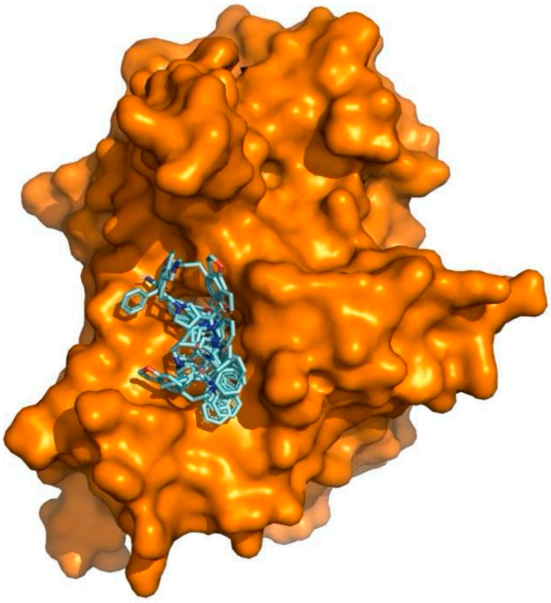

Vina score (kcal/mol) (Darifenacin)	Cavity
-9.2	Other
-8.1	Active site
-7.8	Other
-7.5	Other
-7	Other

Vina score (kcal/mol) (dipeptidyl cyclopropyl nitrile inhibitor 3)	Cavity
-7.8	Other
-7.7	Other
-7.7	Other
-7.5	Active site
-6.3	Other

Supplementary Figure S8. Molecular docking analysis of DF within the catalytic cavity of Cathepsin C. (Left) Surface representation of Cathepsin C showing the different docking conformations predicted for DF within the active-site cleft. The ligand is displayed in multiple orientations to illustrate the preferential occupation of the catalytic cavity and the consistency of ligand accommodation throughout docking simulations. (Right) Comparative table showing the predicted binding affinity values obtained for Cathepsin C complexed with DF and with the co-crystallized inhibitory ligand corresponding to the 6IC7 structure. DF exhibited a more favorable predicted interaction energy (-8.1 kcal/mol) than the reference inhibitor (-7.5 kcal/mol), suggesting greater energetic stabilization within the enzyme's catalytic region. Molecular representations were generated using PyM⊚ L, and docking calculations were performed using the CD-Dock server.
